# Mutation-independent Proteomic Signatures of Pathological Progression in Murine Models of Duchenne Muscular Dystrophy

**DOI:** 10.1074/mcp.RA120.002345

**Published:** 2020-09-28

**Authors:** Tirsa L. E. van Westering, Henrik J. Johansson, Britt Hanson, Anna M. L. Coenen-Stass, Yulia Lomonosova, Jun Tanihata, Norio Motohashi, Toshifumi Yokota, Shin'ichi Takeda, Janne Lehtiö, Matthew J. A. Wood, Samir EL Andaloussi, Yoshitsugu Aoki, Thomas C. Roberts

**Affiliations:** 1 Department of Physiology, Anatomy and Genetics, University of Oxford, Oxford, UK; 2 Department of Oncology/Pathology, Cancer Proteomics Mass Spectrometry, SciLifeLab Stockholm, Karolinska Institutet, Stockholm, Sweden; 3 Department of Paediatrics, University of Oxford, Oxford, UK; 4 Department of Molecular Therapy, National Institute of Neuroscience, National Center of Neurology and Psychiatry (NCNP), Kodaira, Tokyo, Japan; 5 Department of Medical, Genetics, School of Human Development Faculty of Medicine and Dentistry, University of Alberta, Edmonton, Alberta, Canada; 6 MDUK Oxford Neuromuscular Centre, Oxford, UK; 7 Department of Laboratory Medicine, Karolinska Institutet, Huddinge, Sweden

**Keywords:** tandem mass spectrometry, iTRAQ, neurodegenerative diseases, quantification, gene expression, Duchenne muscular dystrophy, dystrophin, mdx, mdx52, proteomics

## Abstract

The absence of the dystrophin protein in Duchenne muscular dystrophy (DMD) results in myofiber fragility and a plethora of downstream secondary pathologies. Although a variety of experimental therapies are in development, achieving effective treatments for DMD remains exceptionally challenging, not least because the pathological consequences of dystrophin loss are incompletely understood. Here we have performed proteome profiling in tibialis anterior muscles from two murine DMD models (*mdx* and *mdx52*) at three ages (8, 16, and 80 weeks of age), all *n* = 3. High-resolution isoelectric focusing liquid chromatography-tandem MS (HiRIEF-LC–MS/MS) was used to quantify the expression of 4974 proteins across all 27 samples. The two dystrophic models were found to be highly similar, whereas multiple proteins were differentially expressed relative to WT (C57BL/6) controls at each age. Furthermore, 1795 proteins were differentially expressed when samples were pooled across ages and dystrophic strains. These included numerous proteins associated with the extracellular matrix and muscle function that have not been reported previously. Pathway analysis revealed multiple perturbed pathways and predicted upstream regulators, which together are indicative of cross-talk between inflammatory, metabolic, and muscle growth pathways (*e.g.* TNF, INFγ, NF-κB, SIRT1, AMPK, PGC-1α, PPARs, ILK, and AKT/PI3K). Upregulation of CAV3, MVP and PAK1 protein expression was validated in dystrophic muscle by Western blot. Furthermore, MVP was upregulated during, but not required for, the differentiation of C2C12 myoblasts suggesting that this protein may affect muscle regeneration. This study provides novel insights into mutation-independent proteomic signatures characteristic of the dystrophic phenotype and its progression with aging.

## Abstract (precis)

In this study we report the highest resolution proteomics analysis performed in dystrophic muscle to date. The use of two mouse models of Duchenne muscular dystrophy, at three different ages, has enabled the identification of mutation-independent protein signatures associated with dystrophic muscle, and the progression of disease pathology.

## Graphical Abstract

**Figure:**
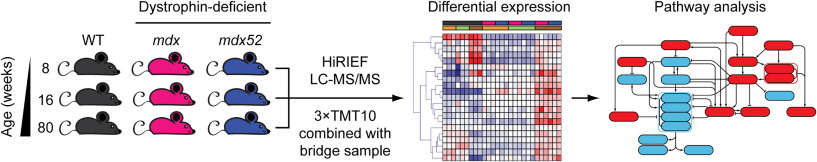

**Highlights**

- Proteomics analysis was performed in two murine models of Duchenne muscular dystrophy (*mdx* and *mdx52*) at three ages (8, 16 and 80 weeks) and compared with wild-type controls.
- High-resolution isoelectric focusing liquid chromatography-tandem mass spectrometry enabled the quantification of 4974 proteins in all samples.
- This study has revealed protein signatures of dystrophin deficiency and the progression of dystrophic pathology.
- In contrast, the proteomes of the *mdx* and *mdx52* mice were highly similar.
- Pathway analysis revealed crosstalk between inflammatory, metabolic and muscle growth processes in dystrophic muscle.

Duchenne muscular dystrophy (DMD) is a severe, X-linked, pediatric neuromuscular disorder characterized by progressive muscle wasting, loss of ambulation around age 10, and cardiorespiratory failure that is ultimately fatal (1–4). The disease is caused by mutations in the *DMD* gene that disrupt the translation reading frame, leading to loss of dystrophin protein expression (5, 6). Dystrophin is important for mechanical force transduction and is also involved in signaling functions (7–11), in part because of its role as an organizing center for the dystrophin-associated protein complex (DAPC). Absence of dystrophin leads to myofiber fragility, sensitivity to contractile damage, chronic cycles of myonecrosis and regeneration, persistent inflammation, and progressive fibro/fatty muscle degeneration (12–14).

The *DMD* gene consists of 79 exons and contains at least seven internal promoters (15) that give rise to the various dystrophin isoforms (*e.g.* Dp427, Dp260, Dp140, Dp116, Dp71, and Dp40) (supplemental Fig. S1). Some isoforms are ubiquitously expressed (*e.g.* Dp71) (16), whereas others are expressed in a more tissue-restricted pattern (such as Dp260, which is the retinal isoform of dystrophin) (17). As a result, the genomic locations of DMD-causing mutations may differentially affect the expression of these various isoforms, and by extension disease manifestation. For example, loss of the Dp71 isoform has been associated with cognitive impairment (18, 19).

**Fig. 1.**
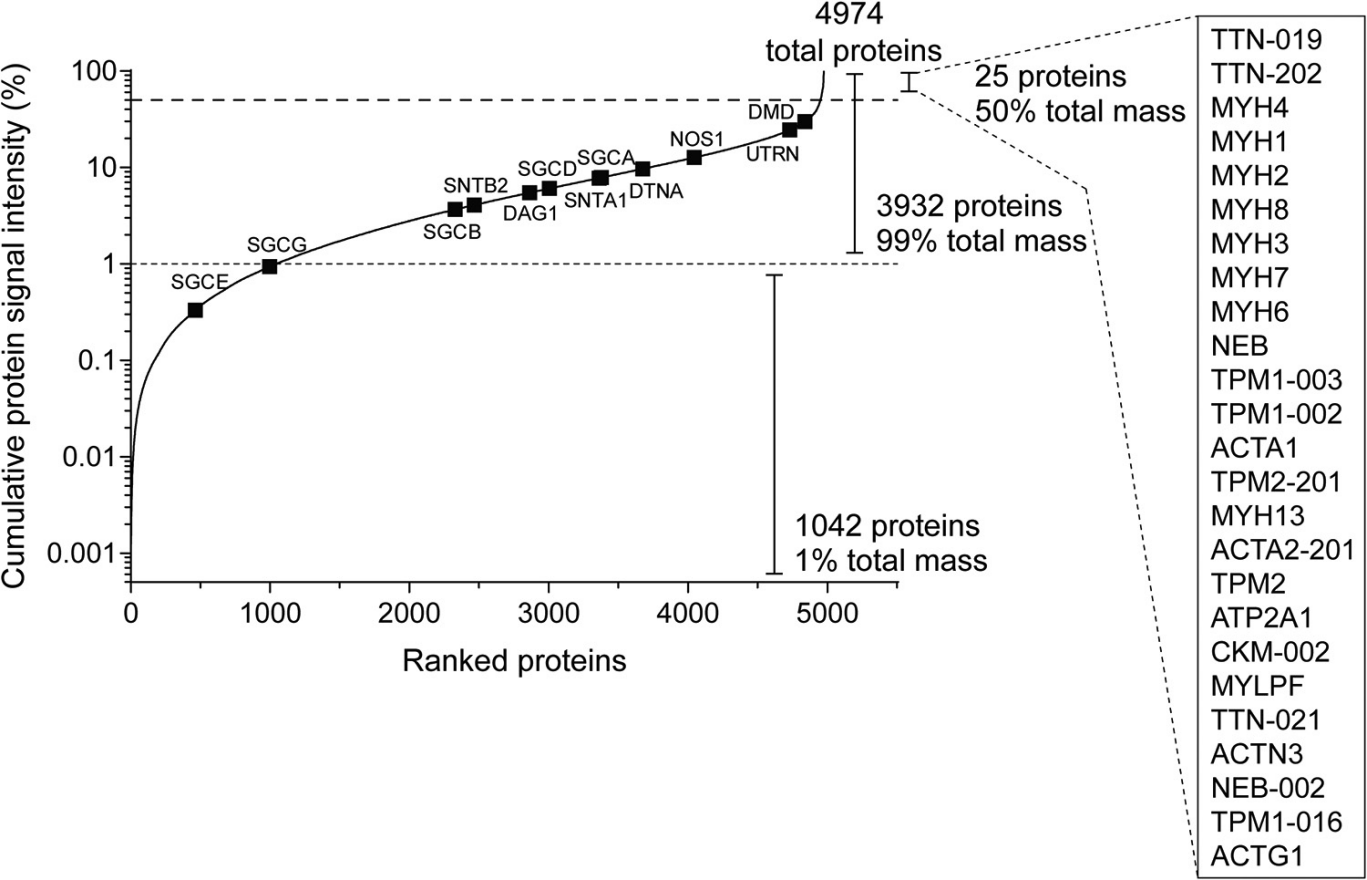
**Protein proportions and dynamic range in murine skeletal muscle tissue.** Peptide-spectrum-match (PSM) data were pooled for all experimental groups for proteins detected in all samples. Proteins were ranked by the number of PSMs/protein and the percentage of total protein signal estimated for each protein by dividing the PSMs/protein by the total number of PSMs, multiplied by 100%. The resulting data are shown as a cumulative frequency plot. Dystrophin-associated protein complex proteins are highlighted as black boxes and the top 25 proteins accounting for 50% of detected protein mass are indicated.

Several dystrophic mouse strains have been developed to investigate DMD pathophysiology, and test novel therapeutics *in vivo* (20). The most used model is the *mdx* mouse, which carries a nonsense mutation in exon 23 leading to loss of the major muscle dystrophin isoform Dp427 (supplemental Fig. S1) (21, 22). Although the *mdx* mouse recapitulates some aspects of DMD pathology (23), it is generally considered to exhibit mild muscular dystrophy with only a small reduction in life-span (24). *mdx* mice undergo a brief period of degeneration and regeneration between 2 and 12 weeks of age (25–27), with more pronounced muscle pathology and cardiomyopathy manifesting much later in life (28, 29). Importantly, the exon 23 mutation observed in the *mdx* mouse does not typically occur in boys with DMD (30), which has motivated the development of more patient-relevant dystrophic mouse models. To this end, Araki *et al.* generated the *mdx52* mouse model in which *Dmd* exon 52 is deleted, leading to the absence of the Dp260 and Dp140 isoforms in addition to Dp427 (supplemental Fig. S1) (31). Deletions in the so-called “hot-spot” region (*DMD* exons 45-55) are some of the most observed mutations in boys with DMD, thereby making this model more patient-relevant than the more widely used *mdx* mouse (6). We recently reported differences in the number of dystrophin-positive revertant fibers and regenerating fibers between *mdx* and *mdx52* mice (32). Specifically, *mdx* mice contained higher numbers of revertant fibers at all ages tested (2–18 months of age), whereas *mdx52* mice contained elevated numbers of centrally-nucleated fibers at 2 months of age only (32). Importantly, the *mdx52* model also allows for the testing of patient mutation-relevant exon skipping strategies *in vivo* (*e.g.* targeting exon 51 or exon 53) (33–35).

Despite significant research effort, many aspects of dystrophic pathology remain unclear. As such, there is a need to understand the complex molecular mechanisms underlying DMD at the level of gene and protein expression. Transcriptomics methodologies have enabled the simultaneous measurement of tens of thousands of genes in the muscles of both dystrophin-deficient animal models and DMD patient biopsy samples (36–40). We have previously compared global transcript and protein expression in *mdx versus* control muscle (39). Importantly, mRNA expression often does not correlate with protein levels (39, 41–43), meaning that investigations that rely on transcriptomics alone may potentially be misleading. Although the global quantification of nucleic acid transcripts is relatively simple using digital gene expression analysis (*i.e.* RNA-sequencing) or hybridization methods (*e.g.* DNA microarrays), proteomics analysis is substantially more challenging. Early proteomics studies in dystrophic muscle used 2D-electrophoresis to identify differentially abundant proteins, but results were limited to only a handful of differential expression calls (44, 45). The use of other methodologies such as iCAT, *in vivo* SILAC, and label-free approaches resulted in the identification of further differentially expressed proteins in dystrophic muscle, although these studies were still limited by their low proteomic coverage (∼1000 proteins quantified, or less) (46–48). Global proteome profiling in fibrous tissues such as skeletal muscle is complicated by the presence of very high concentrations of a few structural proteins, such as actins and myosins (49). Peptides derived from these proteins mask signals from lowly abundant proteins and thereby limit the depth of proteome coverage that can be achieved. To increase analytical depth, we recently applied high-resolution sample pre-fractionation based on narrow-range isoelectric focusing of peptides to quantify expression of over 3272 proteins in mouse muscle (39).

To date, there have been relatively few high-resolution proteomics studies in dystrophic muscle, and fewer still that have measured global changes in protein expression in muscle throughout the progression of pathology over time (39, 47, 50). Here, we have performed MS-based proteomic profiling in both the *mdx* and *mdx52* DMD mouse models compared with WT controls (WT) at three ages representing different stages of dystrophic pathology. We show that this state-of-the-art proteomic strategy has uncovered previously unidentified pathological pathways in dystrophic mouse models, which are potential therapeutic targets for DMD.

## EXPERIMENTAL PROCEDURES

### 

#### 

##### Animals

Mice were housed under 12:12 h light-dark conditions with food and water *ad libitum*. All experimental protocols in this study were approved by the Experimental Animal Care and Use Committee of the National Institute of Neuroscience, NCNP, Japan.

*mdx52* mice were generated at our facility at the NCNP (31) and have been back-crossed with C57BL/6 mice for more than 10 generations. *mdx* mice on a C57BL/6 background were kindly provided by Dr T. Sasaoka (Brain Research Institute, Niigata University, Niigata, Japan). C57BL/6 mice were used as controls to match the background of the dystrophic strains (*i.e. mdx52* and *mdx*). Serum and tissues from each strain were collected at 4, 8, 16, 24, 48, and 80 weeks of age (*n* = 3–5 per group). Tibialis anterior (TA) muscle of all strains at 8, 16, and 80 weeks (*n* = 3) was subsequently cryosectioned, collecting 50 sections of 10 μm for each sample.

##### Cell Culture

C2C12 myoblasts were cultured at 37 °C with 5% CO_2_ in Dulbecco's modified Eagle's medium (DMEM) containing 20% fetal bovine serum (FBS) and 1% antibiotics/antimycotics (growth medium: GM) (all Invitrogen, Carlsbad, CA). DMEM supplemented with 2% horse serum (HS) and 1% antibiotics/antimycotics (differentiation medium: DM) was used to differentiate C2C12 myoblasts for 3–6 days to form multinucleated myotubes. C2C12 myoblasts were seeded in 24-well and 6-well plates at 100,000 cells and 400,000 cells per well respectively. For transfections, cells were incubated in GM before addition of 50 nm siRNA complexes (either targeting *Mvp* or a control siRNA, ON-TARGETplus siRNA: Dharmacon, Cambridge, UK). Complex formation was performed in the absence of serum, and cells collected after 3 days in DM.

##### Serum RNA Extraction and miRNA RT-qPCR

Serum (50 µl) from each sample (*n* = 2–4) was mixed with TRIzol LS (ThermoFisher Scientific), supplemented with 3 µl of a spike-in of 5 nm cel-miR-39 (5ʹ-UCACCGGGUGUAAAUCAGCUUG-3ʹ) per sample as an exogenous reference control. RNA was then extracted according to manufacturer's instructions, with modifications previously described in (51). Samples were stored at −80 °C. TaqMan MicroRNA Reverse Transcription Kit (Applied Biosystems, ThermoFisher Scientific, Waltham, MA) was used for cDNA synthesis of miRNAs. Primers for miR-1a-3p (5ʹ-UGGAAUGUAAAGAAGUAUGUAU-3ʹ), miR-133a-3p (5ʹ-UUUGGUCCCCUUCAACCAGCUG-3ʹ), miR-206-3p (5ʹ-UGGAAUGUAAGGAAGUGUGUGG-3ʹ), miR-223-3p (5ʹ-UGUCAGUUUGUCAAAUACCCCA-3ʹ) and cel-miR-39 were used, following manufacturer's instructions. TaqMan Gene Expression Master Mix (Applied Biosystems) was used for qPCR, following manufacturer's instructions. Primers for miR-1a-3p, miR-133a-3p, miR-206-3p, miR-223-3p and cel-miR-39 were obtained from ThermoFisher and data were normalized as previously described (52).

### HiRIEF-nanoLC-MS/MS-Based Proteomics

#### 

##### Sample Preparation for Mass Spectrometry

Sections of tibialis anterior from C57BL/6, *mdx* and *mdx52*, in biological triplicates, at 8, 16, and 80 weeks were lysed with 4% SDS, 25 mm HEPES, 1 mm DTT. Samples were prepared using a modified version of the spin filter aided sample preparation protocol (53). Lysates were heated to 95 °C for 5 min followed by sonication for 1 min and centrifugation at 14,000 × *g* for 15 min. The supernatant was mixed with 1 mm DTT, 8 M urea, 25 mm HEPES, pH 7.6 and transferred to a 10 kDa cutoff Nanosep centrifugation filtering unit (Pall, Port Washington, NY) for centrifugation at 14,000 × *g* for 15 min. Proteins were alkylated by treatment with 50 mm iodoacetamide in 8 M urea, 25 mm HEPES for 10 min. The proteins were then centrifuged at 14,000 × *g* for 15 min followed by 2 more additions and centrifugations with 8 M urea, 25 mm HEPES. Trypsin (Promega, Southamption, UK) in 250 mm urea, 50 mm HEPES was added to the cell lysate at a ratio of 1:50 trypsin/protein and incubated overnight at 37 °C with gentle shaking. The filter units were centrifuged at 14,000 × *g* for 15 min followed by another centrifugation with Milli-Q water and the flow-through was collected. Peptides were labeled using the TMT10plex Isobaric Label Reagent Set according to the manufacturer's protocol (Thermo Fisher Scientific) and cleaned-up using strata-X-C-cartridges (Phenomenex, Torrance, CA).

##### IPG-IEF of Peptides

TMT labeled peptides were separated by immobilized pH gradient - isoelectric focusing (IPG-IEF) on pH 3.7–4.9 strips (250 µg peptides per strip) as described previously (39, 53). Peptides were extracted from the strips using a prototype liquid handling robot, supplied by GE Healthcare Bio-Sciences AB. A plastic device with 72 wells was put onto each strip and 50 µl of Milli-Q water was added to each well. After incubation for 30 min, the liquid was transferred to a 96-well plate and the extraction was repeated 2 more times. The extracted peptides were dried in speed vac for storage and dissolved in 3% acetonitrile, 0.1% formic acid before MS analysis.

##### LC–MS/MS Analysis

Online LC–MS was performed using a hybrid Q Exactive - HF mass spectrometer (Thermo Fisher Scientific). Before analysis on the Q Exactive (Thermo Fisher Scientific), peptides were separated using an Ultimate 3000 RSLCnano system. Samples were trapped on an Acclaim PepMap nanotrap column (C18, 3 μm, 100 Å, 75 μm x 20 mm), and separated on an Acclaim PepMap RSLC column (C18, 2 μm, 100 Å, 75 μm x 50 cm) (Thermo Fisher Scientific). Peptides were separated using a gradient of A (5% DMSO, 0.1% formic acid) and B (90% acetonitrile, 5% DMSO, 0.1% formic acid), ranging from 6% to 37% B in 50 min with a flow of 0.25 µl/min. The Q Exactive was operated in a data-dependent manner, selecting top 5 precursors for fragmentation by higher-energy collisional dissociation (HCD). The survey scan was performed at 70,000 resolution from 400–1600 *m*/*z*, with a max injection time of 100 ms and a target of 1 × 10^6^ ions. For generation of HCD fragmentation spectra, a max ion injection time of 150 ms and automatic gain control of 1 × 10^5^ were used before fragmentation at 30% normalized collision energy, 35,000 resolution. Precursors were isolated with a width of 2 *m*/*z* and put on the exclusion list for 70 s. Single and unassigned charge states were rejected from precursor selection.

##### Peptide and Protein Identification

All Orbitrap data were searched using SequestHT under the software platform Proteome Discoverer 1.4 (Thermo Fisher Scientific) against the Ensembl 78 mouse protein database (53,838 protein entries) and filtered to a 1% false discovery rate (FDR). A precursor mass tolerance of 10 ppm, and product mass tolerances of 0.02 Da for HCD-FTMS were used. Further settings used were: trypsin with 2 missed cleavage; iodoacetamide on cysteine and TMT on lysine and N-terminal as fixed modifications; and oxidation of methionine as variable modification. Quantification of TMT-10plex reporter ions was performed using Proteome Discoverer on HCD-FTMS tandem mass spectra using an integration window tolerance of 10 ppm. Only peptides unique to a protein group were used for quantitation. A pool of all samples was used in one TMT tag as a linker (denominator) between TMT sets. For data analysis, proteins that could not be identified in all samples were filtered out from the final list and values were log_2_ transformed.

##### Western Blot

Tissue samples were lysed in 75 mm Tris-HCL (pH 6.8), supplemented with 10% SDS, 5% 2-mercapto-ethanol and 3% protease inhibitors (Sigma-Aldrich, Dorset, UK). These were then heated at 100 °C for 3 min and centrifuged at 13,000 × *g* for 10 min. Cells were lysed in RIPA lysis buffer (ThermoFisher Scientific) supplemented with protease and phosphatase inhibitors (Sigma-Aldrich), incubated at 4 °C for 5 min, then centrifuged at 10,000 × *g* for 5 min. Protein was stored at −80 °C. Protein lysates were resolved on polyacrylamide gels and transferred onto 0.45 μm polyvinylidene fluoride (PVDF) membranes. Membranes were blocked in TBST (50 nm Tris pH 7.5, 150 mm NaCl and 0.1% Tween 20), supplemented with 5% nonfat dry milk for 1 h at room temperature. These were subsequently probed with the primary antibody in TBST/blocking solution overnight at 4 °C. Membranes were then incubated with the secondary antibody for one hour at room temperature. Membranes were imaged using the ECL Prime Western blotting system (Sigma-Aldrich) and imaged using an Odyssey Fc Imager (LI-COR Biotechnology, Lincoln, NE). Antibodies and their dilutions are detailed in supplemental Table S1. Equal protein loading was determined by Coomassie Brilliant Blue (CBB) staining for tissue samples and GAPDH immunoblot for cell culture samples was used to determine protein loading.

##### RT-qPCR

RNA was extracted using TRIzol reagent according to manufacturer's instructions. Briefly, samples were homogenized in TRIzol (Thermo Fisher Scientific) after which chloroform was added. Samples were centrifuged resulting in phase separation whereby the supernatant was removed and RNA precipitated with 500 µl of isopropanol, followed by a 10 min incubation at room temperature and centrifugation. Pellets were subsequently washed with 75% ethanol, air-dried and resuspended in nuclease-free water. RNA was stored at −80 °C.

High-capacity cDNA Reverse Transcription Kit (Thermo Fisher Scientific) was used for cDNA synthesis according to manufacturer's instructions. Power SYBR Green master mix was used for gene expression analysis according to manufacturer's instructions, using 1:10 diluted cDNA (primer sequences can be found in supplemental Table S2). Values were normalized to the geometric average of two stable reference genes, *Rplp0* and *Tbp* for tissue samples and *Rplp0* for cells. RT-qPCR data were analyzed using the Pfaffl method and PCR efficiencies determined by LinRegPCR (Amsterdam Medical Center, Amsterdam, the Netherlands).

##### Immunofluorescence

Cells were fixed in 4% paraformaldehyde for 10 min and permeabilized for 15 min in 0.2% Triton X-100. Cells were then blocked for 30 min in 5% Bovine Serum Albumin and incubated with anti-MHC antibody at a 1:20 dilution (MF20, obtained from the Developmental Studies Hybridoma Bank (DSHB), created by the NICHD of the NIH and maintained at The University of Iowa, Department of Biology, Iowa City, IA) overnight at 4 °C. Cells were subsequently probed with secondary antibody (1:500 dilution, goat anti-mouse AlexaFluor 488) and Hoechst33342 (1:5,000 dilution, Thermo Fisher Scientific) for 1 h at room temperature, then stored at 4 °C until imaged. Myogenic Index and Fusion Index were defined as the percentage of nuclei within MHC-positive cells, and the percentage of nuclei within MHC-positive myotubes containing at least 3 nuclei, respectively.

##### Statistics and Bioinformatics

Statistical analysis was performed using R i386 3.2.0 (The R Project) and R Studio (Boston, MA). TMT ratios were log_2_ transformed and significance was tested by *t* test for two-sample comparisons and one-way ANOVA for comparisons between more than two groups using the *rowttests* (genefilter library) and *row.oneway.anova* (HybridMTest library) R functions respectively. *P*-values were corrected for multiple comparisons using the Benjamini-Hochberg method with the *p.adjust* function from the base R. A stringent adjusted *P*-value threshold of 0.01 was used to limit the number of false discoveries. Further statistical analysis on individual proteins was performed with GraphPad Prism 7 (GraphPad Software Inc, La Jolla, CA), whereby one-way ANOVA comparisons were made at each age with Tukey *post hoc* correction. PCA analysis was performed using the *prcomp* R function. GraphPad Prism 7 was used for visualizing PCA, volcano, and other plots. TMeV (Institute for Genomic Research, Rockville, MD) (54) was used to generate heatmaps and identify *k*-means protein clusters. The optimum number of k-clusters was determined using Figures of Merit in TMeV and the elbow method using the *wssplot* R function. *k*-means plots were generated in R.

Gene list enrichment analysis was performed using ToppFun (55). Canonical pathway analysis and upstream regulator analysis was performed using Ingenuity Pathway Analysis (IPA, Qiagen Bioinformatics, Redwood City, CA). *P* < 0.01 and Z≥× 2 × were considered significant. Venn diagrams were generated using Venny 2.1.0 (BioinfoGP Service, Centro Nacional de Biotechnología (CNB-CSIC), Madrid, Spain).

## RESULTS

### 

#### 

##### Experimental Design

To investigate differential protein expression in dystrophic muscle we collected tibialis anterior (TA) muscles from two dystrophic mouse models (*mdx* and *mdx52*). Muscles were collected from age- and sex-matched C57BL/6 WT controls (all mice in this study were male). The choice of ages was based on our previous observations of differences in muscle regeneration and the number of revertant fibers between the *mdx* and *mdx52* strains (32), and on the analysis of serum miRNA levels (supplemental Fig. S2). Our group and others have shown that the muscle-enriched microRNAs (the myomiRs: miR-1a-3p, miR-133a-3p, and miR-206-3p) are highly elevated in serum of dystrophic animal models and DMD patients, indicative of increased muscle turnover (56–62). We therefore analyzed serum myomiR expression at different ages throughout the course of dystrophic pathology to characterize myomiR abundance patterns in the two dystrophic mouse models with age. Serum from animals at 4, 8, 16, 24, 48, and 80 week-old mice was collected, RNA extracted, and myomiRs measured by small RNA TaqMan RT-qPCR. miR-223-3p was included as a nonmyomiR endogenous control that was expected to be relatively stable based on previous studies (52, 56, 63). MyomiR expression was elevated in *mdx* and *mdx52* relative to WT at all ages (supplemental Fig. S2). Interestingly, myomiR levels in *mdx52* serum peaked earlier than for the *mdx* mice (*i.e.* 8 weeks *versus* 16 weeks), consistent with the earlier peak of muscle regeneration observed in this model (32). At the later ages, myomiR abundance remained significantly increased relative to controls. Based on these findings, we selected the 8, 16, and 80-week ages in which to perform proteomic profiling across the mouse strains. The 80-week samples were included as a representative of “aged” muscle in which dystrophic pathology is more advanced (12, 58).

**Fig. 2.**
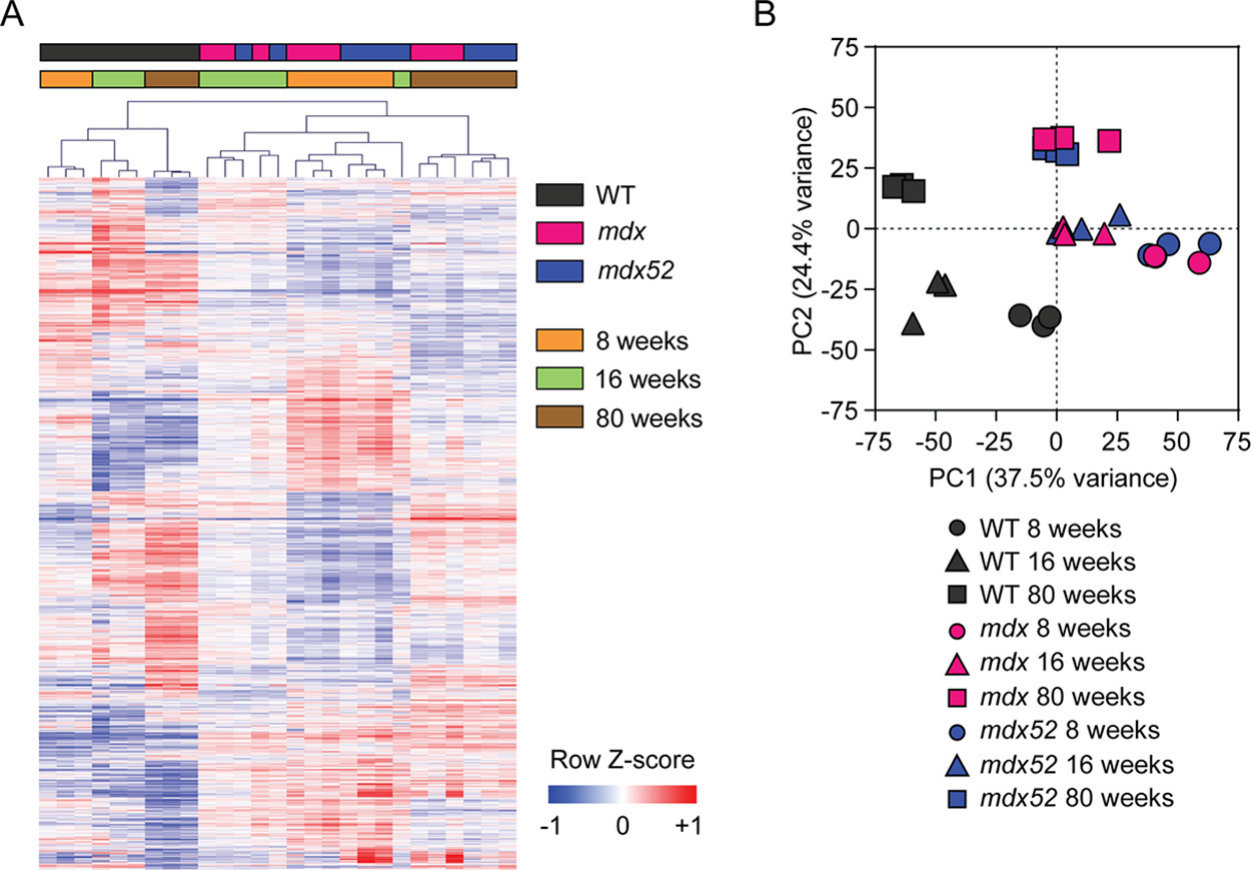
**Differential protein expression in dystrophic muscle.** Quantitative proteomics analysis of TA muscles from WT (C57BL/6), *mdx* and *mdx52* mice at 8, 16 and 80 weeks of age (all *n* = 3). Significantly different proteins identified by one-way ANOVA (Benjamini-Hochberg adjusted *P* < 0.01) are visualized by (*A*) hierarchical clustering and heatmap, and (*B*) principal component analysis (PCA). Equivalent analyses for all proteins are shown in supplemental Fig. S4.

Using these samples, we aimed to identify differences between the *mdx* and *mdx52* models that may account for their distinct phenotypes, establish a mutation-independent proteomic signature of the dystrophic condition, and to investigate alterations in the dystrophic proteome associated with pathological progression.

##### Proteomics Analysis in Dystrophic Muscle

TA protein lysates from male C57BL/6, *mdx*, and *mdx52* mice at 8, 16, and 80 weeks of age (*n* = 3) were analyzed by HiRIEF-LC–MS/MS (53) and samples labeled using TMT chemistry, allowing for multiplexing of up to 10 samples during a single LC–MS/MS run. Three separate runs were performed whereby a common bridge sample (generated by mixing protein lysates from each sample in equal amounts) was included in each set of 10 samples. TMT ratios were calculated relative to the bridge sample, thereby allowing for direct comparison of samples analyzed on different runs, and effectively increasing the level of multiplexing to 27-plex.

A total of 7111 proteins were identified, of which 4974 (70%) were quantified in all samples and used for proteomics analysis (Fig. 1). Details of peptides analyzed for protein identification and quantification are shown in supplemental Fig. S3. Notably, methodological performance was highly similar when comparing between the three separate LC–MS/MS runs (supplemental Fig. S3*B*). This performance is a substantial improvement on our previous data using similar methodology (iTRAQ labeling with HiRIEF-LC–MS/MS, where 3057 proteins were quantifiable (39)). Normalized TMT ratios for the full data set are provided in supplemental File S1.

**Fig. 3.**
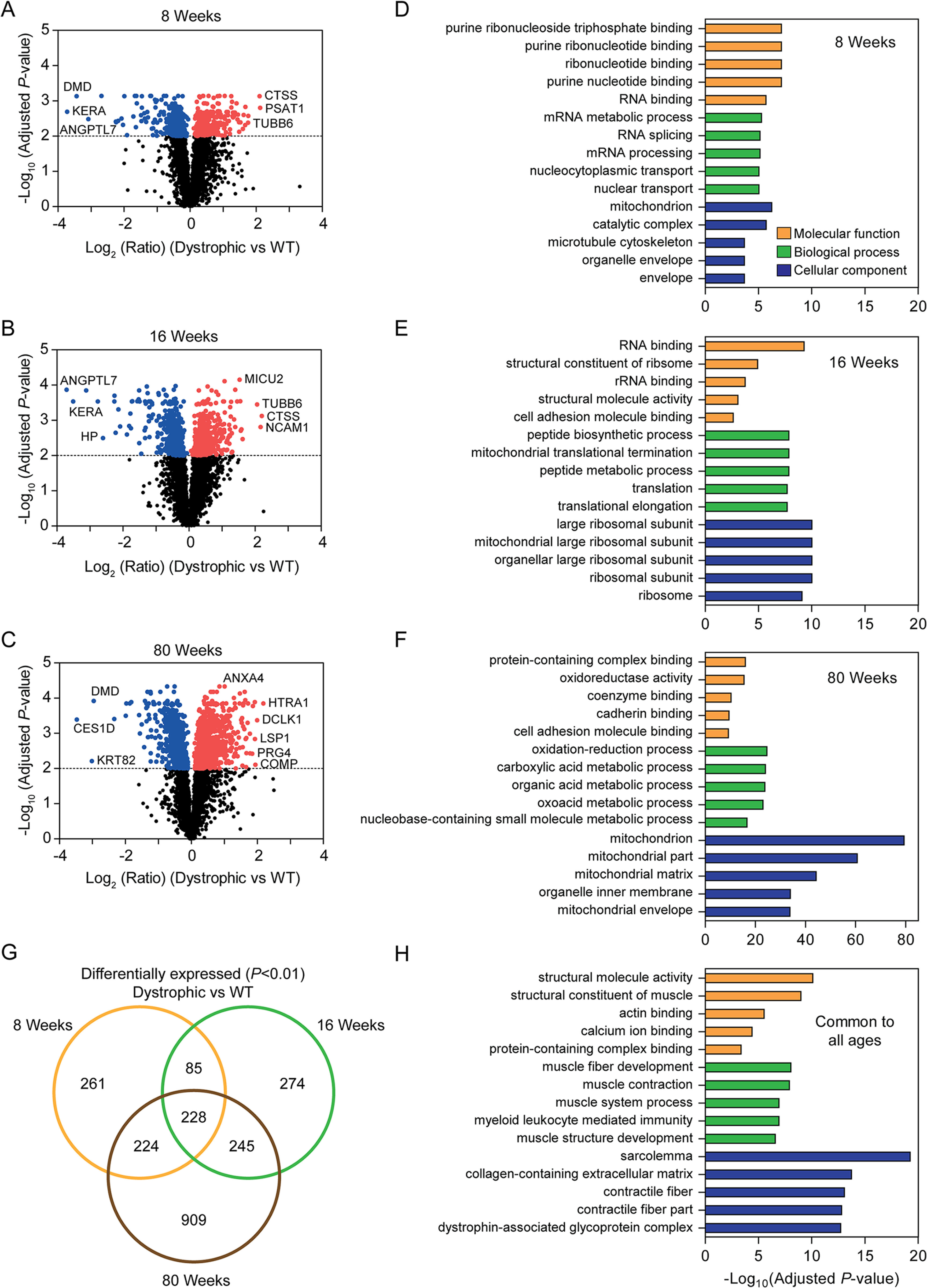
**Differentially expressed proteins in pooled dystrophic muscle samples at 8, 16 and 80 weeks of age.** TMT ratios for the *mdx* and *mdx52* samples (Dystrophic) were pooled and differential expression relative to WT controls assessed at each age (Student's *t* tests, Benjamini-Hochberg adjusted *P* < 0.01). Volcano plots for (*A*) 8 weeks, (*B*) 16 weeks, and (*C*) 80 weeks of age. Gene list enrichment analysis was performed for proteins that were uniquely differentially expressed at (*D*) 8 weeks only, and (*E*) 16 weeks only. *G*, Venn diagram of differentially expressed proteins (dystrophic *versus* WT) showing the overlap between ages. *H*, Gene list enrichment analysis in proteins that were commonly differentially expressed at all ages.

Analysis of the protein cumulative distribution frequency showed that only 25 proteins accounted for 50% of the detected protein mass, which included myosin heavy chain proteins (MYH1, MYH2, MYH3, MYH4, MYH6, MYH7, MYH8, and MYH13), actins (ACTA1, ACTA2), actinin (ACTN3), titin (TTN), nebulin (NEB), tropomyosin (TPM1), and creatine kinase (CKM) (Fig. 1). In contrast, the least abundant 1042 proteins comprised only 1% of the matched spectra (Fig. 1).

Proteins that differ between any of the nine experimental groups were identified by one-way ANOVA analysis and 3492 proteins (70.2%) were identified as significantly different (Benjamini-Hochberg adjusted *P* < 0.01). TMT ratios for differentially expressed proteins were visualized by heatmap with unsupervised hierarchical clustering (Fig. 2*A*) and principal component analysis (PCA, Fig. 2*B*). Biological replicates were tightly clustered, and a clear separation between the WT animals and the two dystrophic strains was apparent. *mdx* and *mdx52* samples were intermingled, suggestive of a common pattern of proteomic perturbation in these dystrophic models. The greatest source of variation in the data, represented by principal component 1 (PC1, containing 37.5% of the variation) on the PCA biplot (Fig. 2*B*), primarily reflected the difference between WT and dystrophic animals. Conversely, principal component 2 (PC2, containing 24.4% of the variation) reflected the progression of age, with the low, medium, and high values of PC2 corresponding to 8-, 16-, and 80-week-old mice, respectively (Fig. 2*B*). Highly similar clustering patterns were observed when these analyses were performed on all proteins (supplemental Fig. S4).

**Fig. 4.**
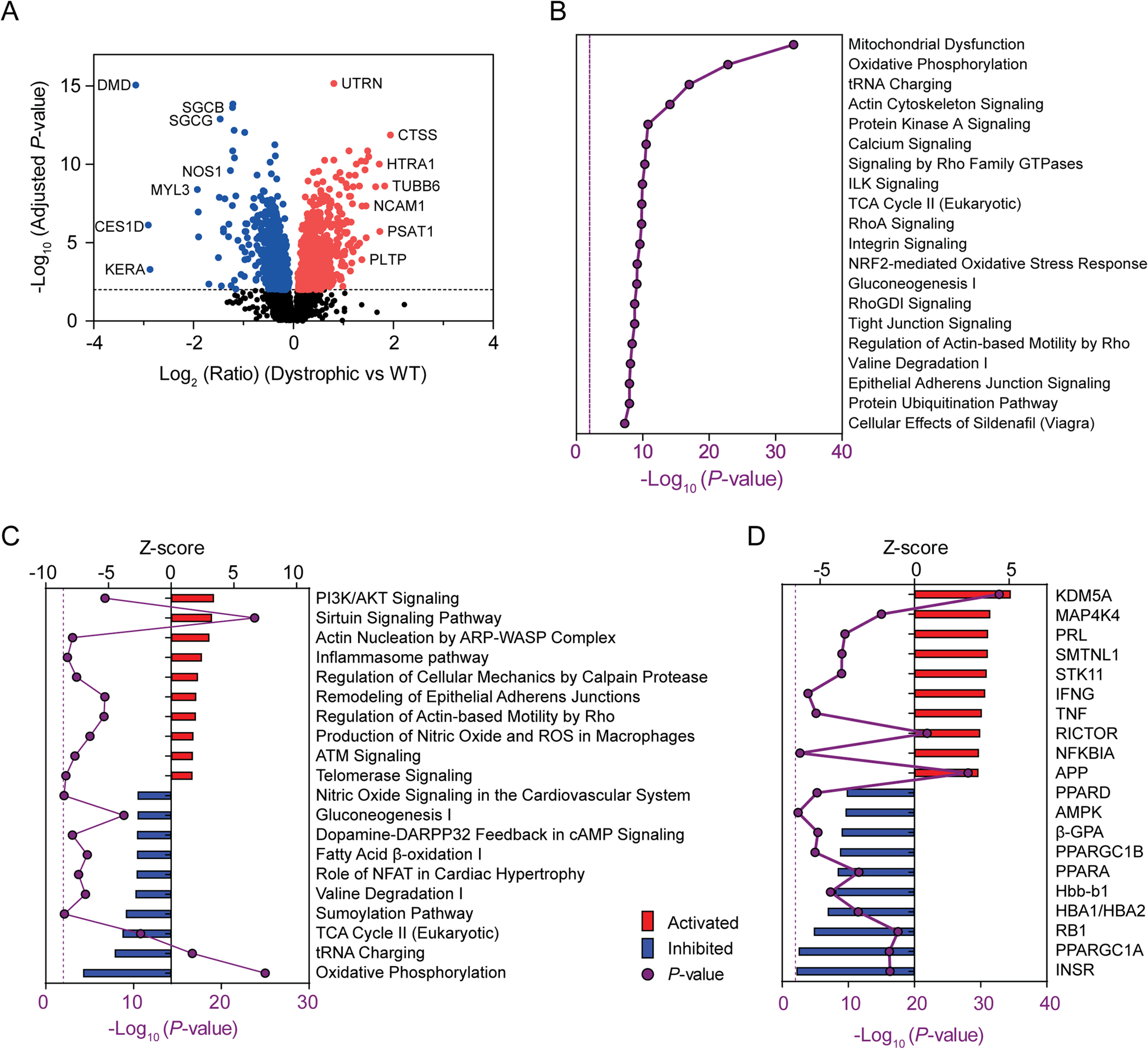
**Differentially expressed proteins and pathway analysis in dystrophic muscle independent of age or mutation type.** All TMT ratios for the *mdx* and *mdx52* samples (Dystrophic, *n* = 18) were pooled and differentially expressed proteins determined relative to WT controls (*n* = 9) independent of age (Student's *t* tests, adjusted *P* < 0.01). Differentially expressed proteins were (A) visualized by volcano plot and analyzed with IPA in order to identify (B) the top 20 most significant (*P* < 0.01) enriched canonical pathways, (C) the top 10 up- and downregulated pathways based on activation state (*i.e.* Z-score), and (D) putative upstream regulators (based on the expression ratios of their known downstream targets). Canonical pathways and upstream regulators are ranked by Z-score (positive values indicate activation and negative values indicate inhibition of the predicted regulator). Solid purple lines indicate *P*-value. Broken purple lines indicate the *P* = 0.01 significance threshold.

To assess the performance of the proteomics analysis we examined the expression of proteins that are known to be differentially expressed in dystrophic muscle. Dystrophin (DMD) was observed to be highly downregulated in the dystrophic animals at all ages, consistent with its genetic disruption. Accordingly, expression of the DAPC components: NOS1, SGCA, SGCB, SGCG, DAG1, SNTA1, and DTNA were all significantly downregulated in both *mdx* and *mdx52* mice at all ages (supplemental Fig. S5). Loss of dystrophin leads to the mislocalization and subsequent destabilization of DAPC components (64). As a result, downregulation of DAPC proteins is an established feature of dystrophic muscle (39, 65). In contrast, UTRN (utrophin) was significantly upregulated in dystrophic muscle (supplemental Fig. S5). Utrophin is a paralog of dystrophin that is primarily expressed at the neuromuscular and myotendinous junctions and is known to be upregulated at the dystrophin-deficient sarcolemma (39, 66–69). Similarly, aquaporin-4 (AQP4), periostin (POSTN), myostatin (MSTN), and biglycan (BGN) were also differentially expressed in dystrophic muscle as reported previously (39, 70–73). In summary, consistent differential expression of these proteins in both dystrophic strains across all ages (supplemental Fig. S5) demonstrates the robustness of both our proteomics analysis and multiplexing strategy.

**Fig. 5.**
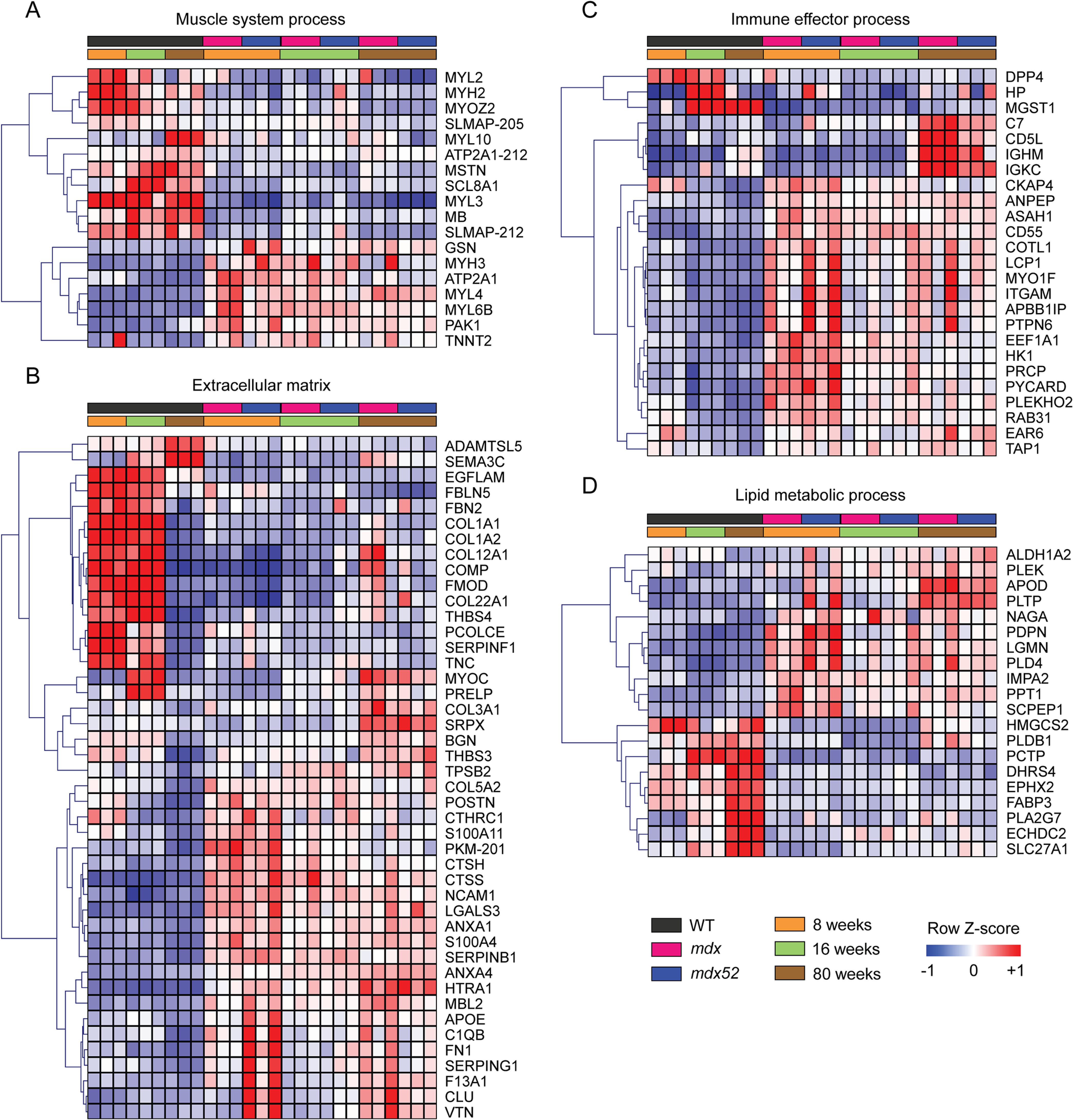
**Categorization of the most differentially expressed proteins in dystrophic muscle**. Proteins that were significantly different (adjusted *P* < 0.01) in at least one inter-sample comparison with a fold change greater than 2 (excluding the DAPC components: DMD, DAG1, DNTA, NOS1, SNTA1, SGCA, SGCB, SGCG, SGCD) were analyzed using gene list enrichment analysis. Protein expression was visualized by heatmap for: *A*, muscle system process (GO:0003012), *B*, extracellular matrix (GO:0031012), *C*, immune effector process (GO:0002252), and *D*, lipid metabolic process (GO:0006629).

##### A Mutation-Independent Dystrophic Proteome Signature

No significant changes in protein expression were detected when comparing the *mdx* and *mdx52* dystrophic strains by *t* test (supplemental Fig. S6*A*), consistent with the close clustering of these samples in the heatmap and PCA analyses described above (Fig. 2). Absolute fold changes for the *mdx52 versus mdx* comparisons were lower than for other comparisons (supplemental Fig. S7*A*), and the coefficients of variation for the *mdx52* samples are similar to, or lower than, other experimental groups (indicating that these samples are not inherently noisy, supplemental Fig. S7*B*). Together these data suggest that the lack of significant differential expression calls in the *mdx52 versus mdx* comparisons is a consequence of the high similarity of the muscle proteomes in these strains, rather than a deficiency in statistical power.

**Fig. 6.**
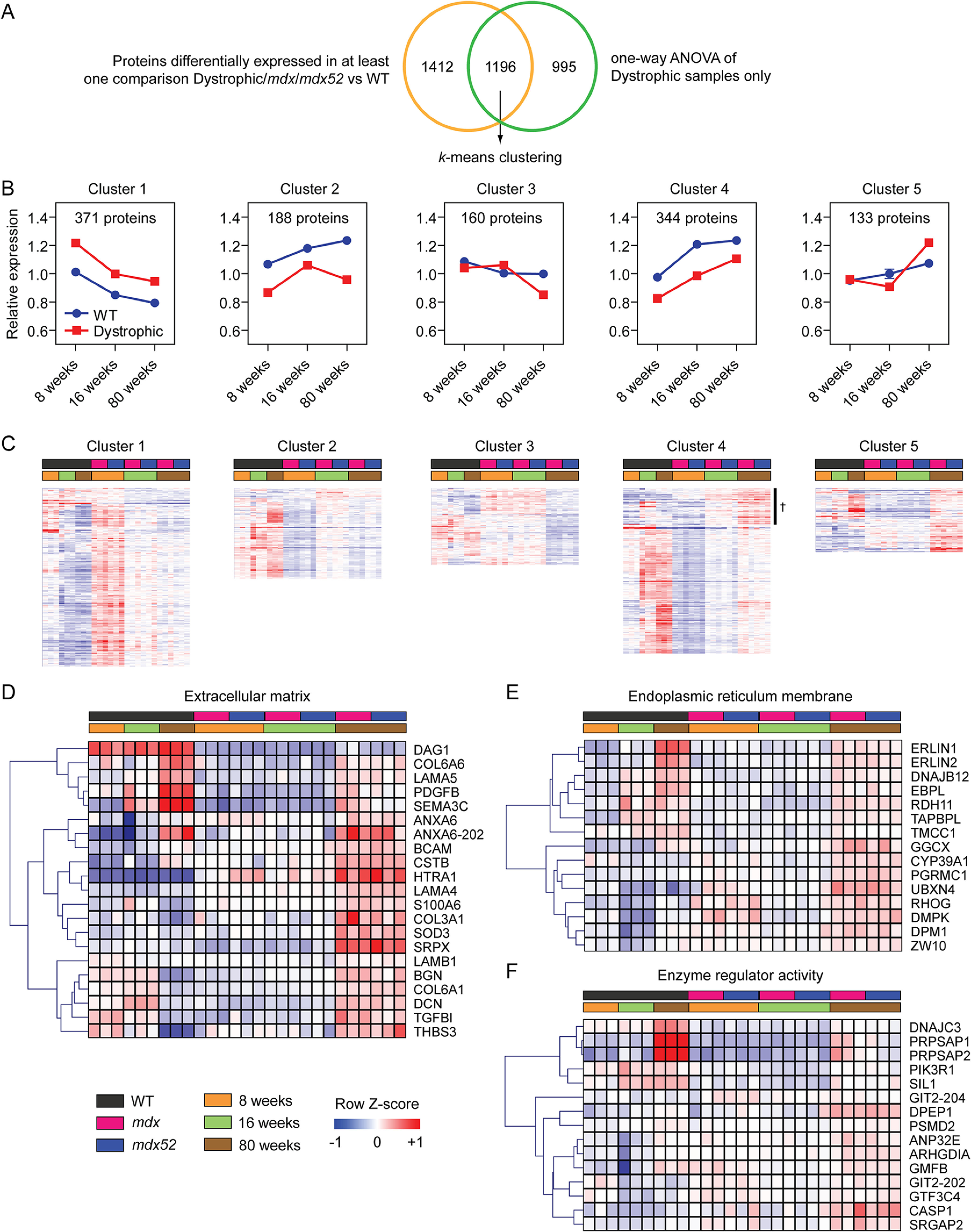
**Changes in dystrophy-associated protein expression with pathological progression.** *A*, Venn diagram showing the overlap between significantly different (adjusted *P* < 0.01) proteins when comparing all the Dystrophic *versus* WT comparisons and the 8-week *versus* 16-week *versus* 80-week comparisons (performed in the Dystrophic samples only). The resulting 1,196 proteins were analyzed using *k*-means clustering. *B*, Line plots showing the mean±S.E. expression values across all proteins contained in each *k*-cluster. *C*, Expression for individual proteins contained in each cluster is shown by heatmap. A subset of proteins in cluster 4 that are upregulated in 80 week dystrophic muscle only are highlighted with the † symbol. Cluster 5 was further analyzed, and protein expression was visualized by heatmap for proteins associated with the following gene ontology terms: *D*, extracellular matrix (GO:0031012), *E*, endoplasmic reticulum membrane (GO:0005789), and *F*, enzyme regulator activity (GO:0030234). All heatmaps were sorted using hierarchical clustering in the gene dimension only, for clarity.

**Fig. 7.**
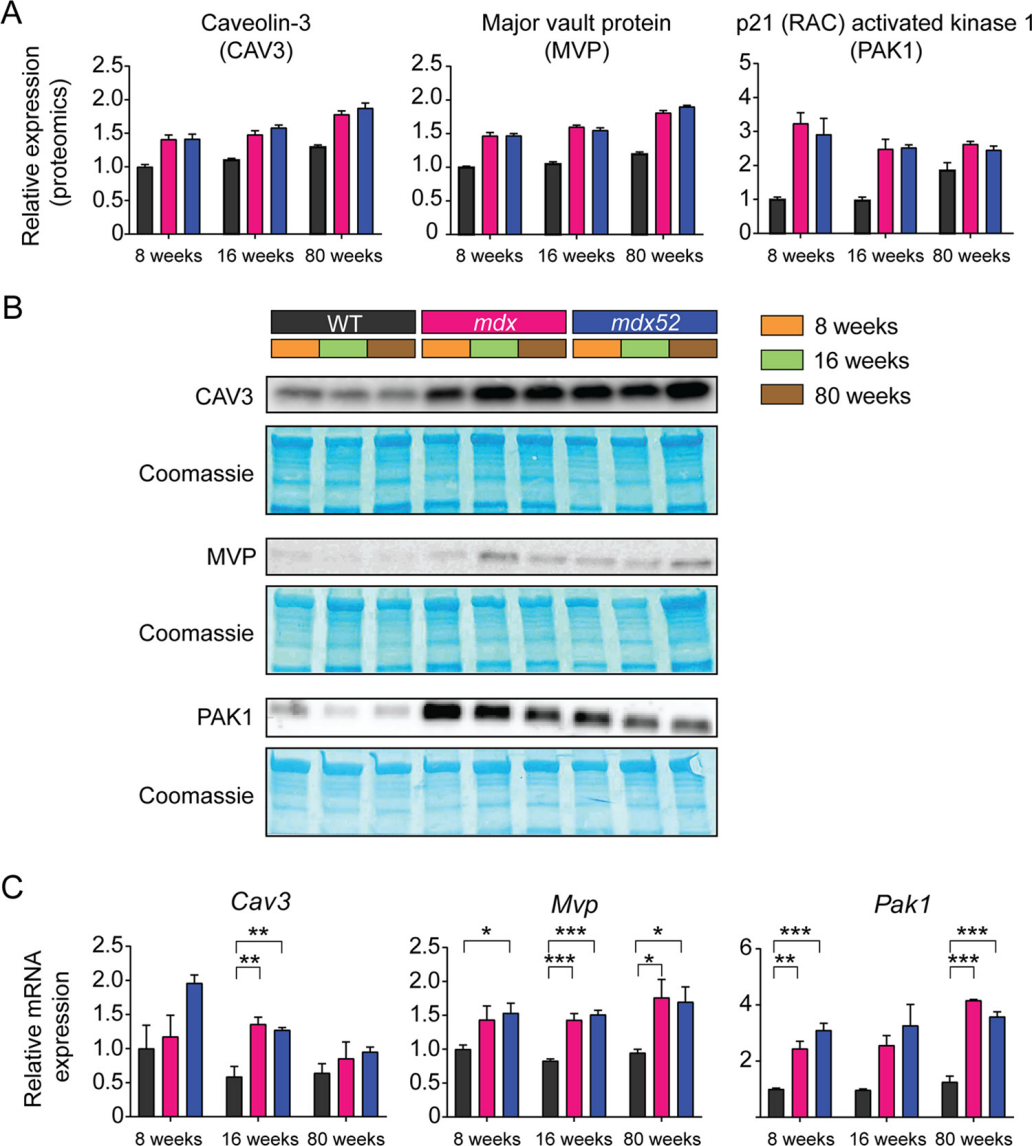
**Validation of CAV3, MVP and PAK1 upregulation in dystrophic muscle.** Relative protein expression of CAV3, MVP and PAK1 was determined by (*A*) HiRIEF-LC–MS/MS, and (*B*) Western blot. Protein loading was assessed by Coomassie Brilliant Blue (CBB) staining. *C*, Relative mRNA expression of *Cav3*, *Mvp* and *Pak1* as determined by RT-qPCR. Genes-of-interest expression was normalized to the geometric mean of two stable reference genes: *Rpl10* and *Tbp*. Statistical analyses are one-way ANOVA at each age, with Tukey *post hoc* correction. All values are mean+S.E., *n* = 3 for protein analysis and *n* = 3-4 for RNA analysis, *P < 0.05, **P < 0.01 and ***P < 0.001.

Separate *t*-tests were performed to compare *mdx versus* WT (supplemental Fig. S6*B*) or *mdx52 versus* WT at each age (supplemental Fig. S6*C*). Relatively few differentially expressed proteins (<35) were observed at the 8 week and 16 week ages. In contrast, several hundred differentially expressed proteins were identified in the 80-week muscle samples, of which 199 were common between the *mdx* and *mdx52* strains (supplemental Fig. S6*D*).

Considering the similarity in proteomes between the two dystrophic strains, and the relatively small number of differences detected when comparing each dystrophic model against the WT controls, we decided to pool the TMT ratios for the *mdx* and *mdx52* samples at each age in order to increase group size (to *n* = 6) and thereby boost statistical power. Differentially expressed proteins in the pooled dystrophic muscles were assessed by *t* test at each age and 805, 837, and 1618 differentially expressed (adjusted *P* < 0.01) proteins identified at 8, 16, and 80 weeks respectively (Fig. 3*A*–3*C*). Proteins that were uniquely upregulated at 8 weeks and 16 weeks were enriched for RNA processing and ribosome/translation-associated gene ontology (GO) terms respectively (Fig. 3*D*, 3*E*). 909 proteins were differentially expressed at 80 weeks only and were highly enriched for GO terms associated with mitochondria and metabolic processes (Fig. 3*F*). 228 proteins were commonly differentially expressed at all three ages (Fig. 3*G*), which were enriched for GO terms associated with muscle function, sarcolemma, extracellular matrix, and the DAPC (Fig. 3*H*).

TMT ratios for all dystrophic (*n* = 18) and all WT (*n* = 9) animals were further pooled, and statistically significant differences between these groups tested by *t* test. The purpose of this analysis was to identify proteins that are differentially expressed in dystrophic muscle independent of mutation type or age (with the added benefit of increased statistical power). Of the 4974 proteins detected, 1795 (36%) proteins were significantly different between pooled dystrophic and WT samples (adjusted *P* < 0.01) (Fig. 4*A*) of which 891 were upregulated and 904 downregulated.

The top 10 consistently up- and downregulated proteins for all dystrophic *versus* WT comparisons are shown in supplemental Figs. S8 and S9 (excluding DAPC component proteins that were highly differentially expressed, as in previous analyses). The most highly upregulated proteins in dystrophic muscle included: AHNAK nucleoprotein 2 (AHNAK2), HtrA serine peptidase 3 (HTRA3), cathepsin S (CTSS), tubulin beta 6 class V (TUBB6), and an expressed pseudogene of carboxylesterase 2D (CES2D-PS). The enzymes SAM decarboxylase 2 (AMD2) and 3-hydroxybutyrate dehydrogenase 1 (BDH1) were among the most downregulated proteins in dystrophic muscle. The proteoglycan keratocan (KERA) was downregulated at the 8 and 16 week ages only. EGF like, fibronectin type III and laminin G domains (EGFLAM, also known as pikachurin) was consistently downregulated in dystrophic muscle and has been previously shown to interact with α-dystroglycan (DAG1) (74) suggesting that downregulation of EGFLAM may be a consequence of DAPC disruption.

**Fig. 8.**
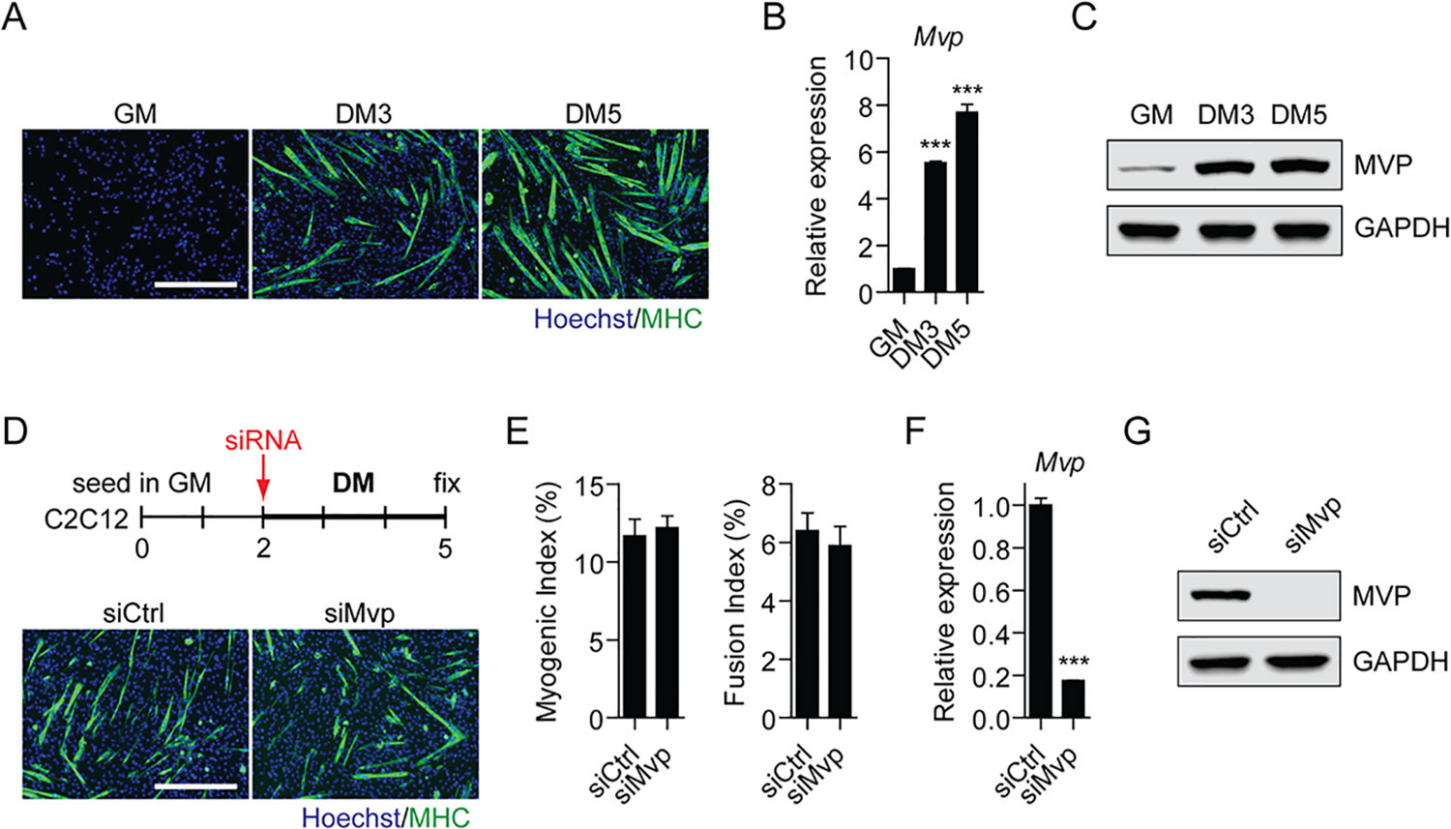
**MVP is upregulated during, but dispensable for, myoblast differentiation.** C2C12 murine myoblasts were cultured in growth media (GM) and then switched to differentiation media (DM) for 3 and 5 days (DM3 and DM5 respectively). *A*, Myogenic differentiation was confirmed by immunofluorescence (IF) staining for myosin heavy chain (MHC). Expression of *Mvp* mRNA was determined by (*B*) RT-qPCR and MVP protein by (*C*) Western blot. C2C12 cells were transfected with 50 nm control (siCtrl) or *Mvp*-targeting (siMvp) siRNAs and fixed after 3 days of culture in DM. Myogenic differentiation was assessed by (*D*) IF staining for MHC and quantified using (*E*) Myogenic and Fusion Indices. Knockdown was confirmed by (*F*) RT-qPCR and (*G*) Western blot. RT-qPCR data were normalized to *Rplp0* expression. GAPDH served as a protein loading control. All values are mean+S.E., *n* = 3 for RT-qPCR and *n* = 4 for IF quantification, *P* < 0.001, one-way ANOVA and Tukey *post hoc* test or student's *t* test as appropriate. Images were taken at 10 × magnification, scale bars indicate 400 μm.

**Fig. 9.**
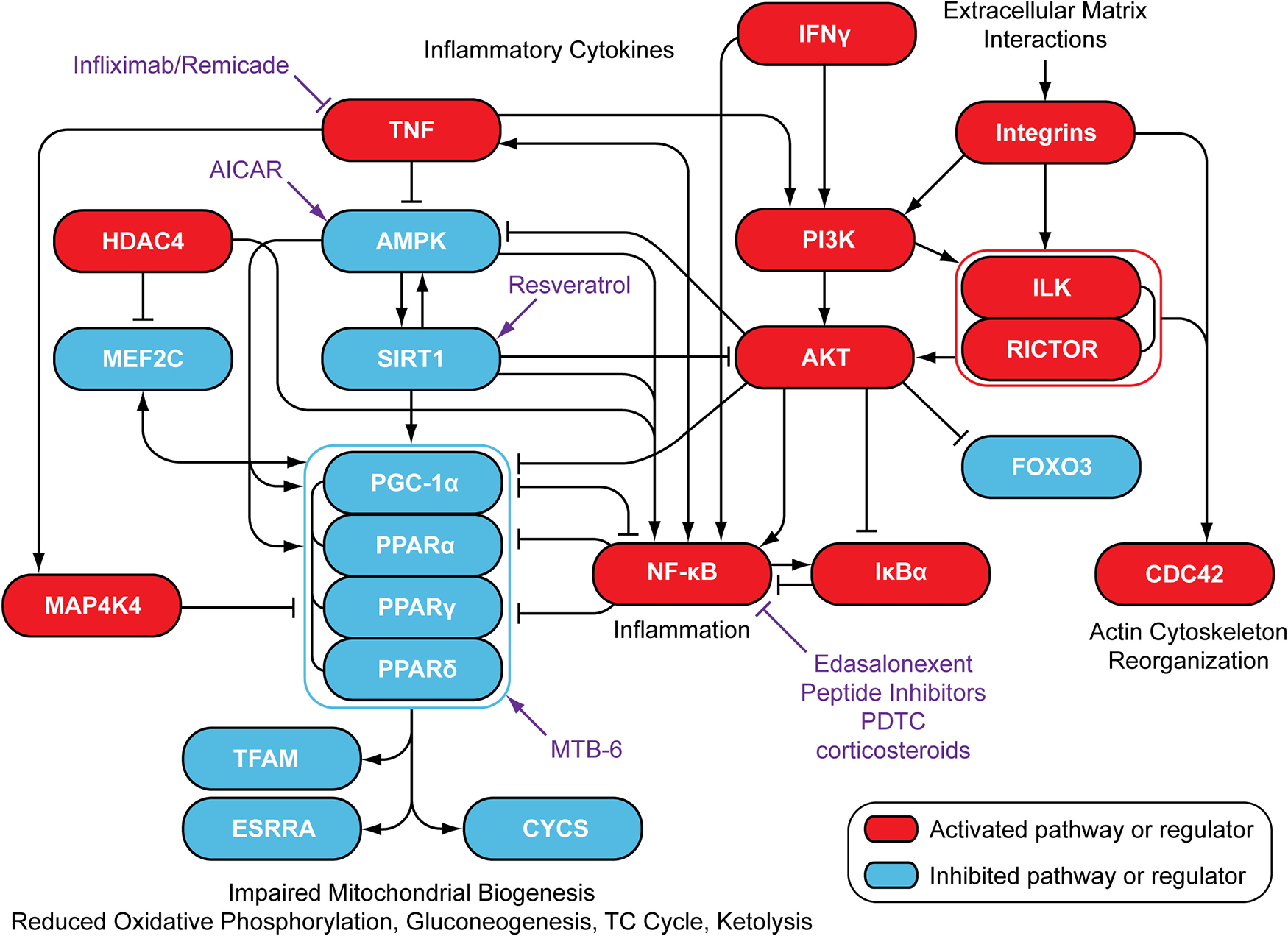
**Cross-talk between metabolic, inflammatory and muscle growth pathways in dystrophic muscle.** Upstream regulator and canonical pathway analyses were integrated to generate an explanatory schema of processes occurring in dystrophic muscle. Drugs with the potential to treat DMD are shown in purple. Activation of NF-κB was not predicted based on our data but has been well-described in dystrophic muscle. ILK Signaling was enriched in dystrophic muscle, although the analysis did not determine an activation state.

Protein expression ratios from the pooled analysis were analyzed using ingenuity pathway analysis (IPA) to identify perturbed canonical pathways (Fig. 4*B*, 4*C*) and predicted upstream regulators (Fig. 4*D*) in dystrophic muscle. This analysis identified multiple findings consistent with established features of dystrophic pathology (*e.g.* calcium signaling, inflammasome pathway, nitric oxide signaling, and TNF signaling). Furthermore, many affected canonical pathways and predicted upstream regulators were identified with no known association with DMD pathology (*e.g.* NRF2-mediated oxidative stress response and ATM signaling, KDM5A, and RICTOR, Fig. 4*B*, 4*C*). Mitochondrial Dysfunction was the most significantly affected canonical pathway, consistent with our previous study (39). Notably, the increased depth of proteome coverage enabled the detection of protein expression changes that were not possible in our previously published analysis (39). For example, multiple protein components of Complex IV were downregulated in dystrophic muscle in addition to the other four complexes of the electron transport chain that we reported previously (39). Metabolic pathways associated with the mitochondria were similarly downregulated (*i.e.* TCA Cycle II, Oxidative Phosphorylation, Fatty Acid β-oxidation I).

To identify the most important proteins contributing to dystrophic pathology, differentially expressed proteins were filtered to include only those with a fold change greater than 2 in at least one comparison, and DAPC members were excluded. The resulting list of 200 proteins was classified into muscle, extracellular matrix, immune response, and lipid metabolism based on gene list enrichment analysis (Fig. 5). Multiple myosin heavy and light chains were differentially expressed in dystrophic muscle (*i.e.* Downregulation of MYH2, MYL2, MYL3, MYL10 and upregulation of MYH3, MYL4, MYL6B), consistent with our previous study (39). Other structural components of muscle were perturbed, including downregulation of myozenin-2 (MYOZ2), myoglobin (MB), the Na^+^/Ca^2+^ exchanger SLC8A1, and two isoforms of sarcolemma associated protein (SLMAP). Conversely, cardiac muscle troponin T (TNNT2), gelsolin (GSN), and p21 (RAC1) activated kinase 1 (PAK1) were upregulated in dystrophic muscle (Fig. 5*A*). In general, differential expression of these muscle-associated proteins was similar across ages, consistent with being consequences of dystrophin absence in general, rather than as downstream pathologies, which may develop with disease progression.

Analysis of differentially expressed extracellular matrix-associated proteins revealed reciprocal regulation of different collagen proteins, with COL1A1, COL1A2, and COL2A1, COL22A1 all downregulated, and collagens COL3A1 and COL5A2 upregulated. (Elevation of collagen-3 has previously been linked to fibrotic pathology in dystrophin-deficient muscle (75)). Other upregulated extracellular matrix-associated proteins included myocilin (MYOC), sushi repeat-containing protein (SRPX), prolargin (PRELP), the serine protease HTRA1, the annexins: ANXA1 and ANXA4, and the S100 proteins: S100A4 and S100A11. Conversely, fibromodulin (FMOD), fibrillin-2 (FBN2), and tenascin C (TNC) were all downregulated in dystrophic muscle. Interestingly, two thrombospondin genes were differentially expressed, although in opposite directions, with THBS3 being upregulated and THBS4 downregulated in dystrophic muscle. Similarly, the serpins: SERPINB1 and SERPINF1, were up and downregulated, respectively. In contrast, with the muscle-associated proteins described above, some extracellular matrix factors were perturbed at the 80-week age only or exhibited a progressive increase in expression with age (*i.e.* COL3A1, MYOC, SRPX, BGN, THBS3, and HTRA1). Together these findings are consistent with widespread matrix remodeling and later-onset fibrotic damage (Fig. 5*B*).

The majority of immune response-associated proteins were upregulated in dystrophic muscle and include macrophage-related genes (integrin alpha M (ITGAM) and CD5L), complement factors (C7), and immunoglobulin genes (IGHM and IGKC) (Fig. 5*C*). Differentially expressed proteins associated with lipid metabolism included fatty acid binding protein 3 (FABP3), phospholipase A2 group VII (PLA2G7), and the long-chain fatty acid transporter SLC27A1, which were downregulated, and apolipoprotein D (APOD), phospholipid transfer protein (PLTP), and legumain (LGMN) that were upregulated in dystrophic muscle (Fig. 5*D*). As with the extracellular matrix-associated proteins, the immune-response and lipid metabolism categories contained some proteins that were differentially expressed at all ages, and some that were perturbed to a greater extent in the 80-week-old samples.

##### Proteomic Changes Associated with the Progression of Dystrophic Pathology

We next sought to identify proteins that were differentially expressed between dystrophic and WT samples, and which exhibited age-associated changes in expression (and therefore pathological progression). One-way analysis of variance was performed on the dystrophic samples in order to identify significant differences (adjusted *P* < 0.01) between 8-, 16-, and 80-week-old pooled dystrophic mice, and this list was then filtered using the list of proteins that were found to be differentially expressed in any of the analyses performed above. The overlapping 1196 proteins were then classified into 5 *k*-clusters based on their expression patterns. The character of each cluster was then illustrated by line graphs of the mean expression of all proteins (Fig. 6*B*) and heatmaps (Fig. 6*C*). Gene list enrichment analysis was performed to assign biological meaning to each cluster.

Cluster 1 contained proteins that progressively declined with age but were generally elevated in dystrophic muscle throughout. This cluster was enriched for GO terms associated with focal adhesion and mRNA splicing. Conversely, Cluster 4 contained proteins that were progressively upregulated with age in both WT and dystrophic muscle. In general, expression of proteins in this cluster was downregulated in dystrophic muscle, although a subset of proteins (marked with † in Fig. 6*C*) were upregulated. This subset included proteins such as integrin linked kinase (ILK), EH domain containing 3 (EHD3), asporin (ASPN), indolethylamine N-methyltransferase (INMT), osteoglycin (OGN), major vault protein (MVP), and immunoglobulin like and fibronectin type III domain containing 1 (IGFN1). Notably, several proteins associated with other muscular dystrophies were also found in this sub-list including caveolin 3 (CAV3), lamin A/C (LMNA), muscleblind like splicing regulator 1 (MBNL1), and fukutin related protein (FKRP) (linked to distal myopathy, Emery-Dreifuss muscular dystrophy, myotonic dystrophy, and limb-girdle muscular dystrophy respectively). The proteins in Cluster 4 were associated with the GO terms sarcomere, sarcolemma, extracellular matrix, neuromuscular junction, and calcium channel complex.

Cluster 2 proteins were downregulated in dystrophic muscle, but the magnitude of downregulation was less apparent in the 16-week-old samples. Proteins in Cluster 3 were downregulated in dystrophic muscle at the 80 week age only. Gene list enrichment analysis showed Cluster 2 and 3 were primarily enriched for mitochondria-related GO terms (data not shown).

Cluster 5 was particularly interesting, as it contained proteins that were upregulated in 80-week-old dystrophic mice, and so are therefore likely associated with more advanced pathological progression. Proteins in Cluster 5 were enriched for the GO terms: extracellular matrix (Fig. 6*D*), endoplasmic reticulum membrane (Fig. 6*E*), and enzyme regulator activity (Fig. 6*F*). The effect sizes for protein expression changes were highest for the extracellular matrix associated changes, which included several proteins discussed above (*i.e.* BGN, COL3A1, HTRA1, SRPX, and THBS3). Notable other extracellular matrix-associated changes in dystrophic muscle included upregulation of annexin A6 (ANXA6), COL6A6, decorin (DCN), platelet derived growth factor subunit B (PDGFB), S100A6, transforming growth factor beta induced (TGFBI), and the laminins: LAMA4, LAMA5, and LAMB1 (Fig. 6*D*). The endoplasmic reticulum membrane-associated changes included upregulation of UBX domain protein 4 (UBX4), gamma-glutamyl carboxylase (GGCX), cytochrome P450 family 39 subfamily A member 1 (CYP39A1), ras homolog family member G (RHOG), and dystrophia myotonica protein kinase (DMPK) (Fig. 6*E*). The enzyme regulator activity-associated changes include upregulation of phosphoinositide-3-kinase regulatory subunit 1 (PIK3R1) and caspase-1 (CASP1).

In summary, the proteins in Clusters 1 and 4 might be considered factors that are differentially expressed with aging, but to a greater magnitude in dystrophic muscle. Conversely, Clusters 3, 4, and 5 represent proteins that are progressively differentially expressed in dystrophic muscle with more advanced pathology.

##### Pathway Analysis in Dystrophic Muscle throughout Pathological Progression

Dystrophic *versus* WT protein expression ratios at each age were analyzed separately using IPA in order to identify changes in canonical pathway and upstream regulator status associated with disease progression. Overall results were like those described above (where statistical power was greater on account of larger sample sizes) (Fig. 4*C*). Many canonical pathways were commonly perturbed across the different ages such as TNFR1 Signaling, PI3K/AKT Signaling, Inflammasome Pathway, Calcium Handling, Nitric Oxide Signaling in the Cardiovascular System (supplemental Fig. S10). However, other pathways exhibited apparent age-associated changes in perturbation. For example, pathways including TWEAK Signaling, Sirtuin Signaling Pathway, Phospholipase C Signaling, Signaling by Rho Family GTPases, and Integrin Signaling were upregulated at all ages but increased their degree of activation at 80 weeks. Conversely, multiple metabolism-associated pathways became progressively inactivated with disease progression, including: Ketolysis, Ketogenesis, Fatty Acid Activation, Gluconeogenesis I, and Fatty Acid β-oxidation I (among others). Pathways associated with tRNA Charging and amino acid degradation were also inhibited at 80 weeks. Interestingly, two pathways, Synaptogenesis Signaling Pathway and GP6 Signaling Pathway, exhibited marked reversals in their activation status (*i.e.* inactivated at 8 and 16 weeks, and activated at 80 weeks) (supplemental Fig. S11).

Similar findings were observed when considering predicted upstream regulators at each age (supplemental Fig. S12). Many of the predicted regulators identified were common to the pooled analysis described above (Fig. 4*D*), including IFNG, TNF, RICTOR, KDM5A, MAP4K4, INSR, TFAM, AMPK, PPARGC1A, PPARA, PPARG, and PPARD. Aging-associated changes in predicted upstream regulators were observed for TGFB1, SEMA7A, PLIN5, IKBKG, DNMT3A and TWIST1 that were activated with age, and MYC, ALDH1A2, VEGFB, TRIM24, and CPT1C that were inhibited with age (among many others) (supplemental Fig. S12). Interestingly, a number of pathways and upstream regulators were differentially modulated to a lesser extent at the 16 week age, relative to the 8 and 80 week ages (supplemental Figs. S11, S12). Such a pattern of expression is also apparent for some proteins in the heatmaps shown in Figs. 5*B*–6*D* and 6*C*, Cluster 2. These patterns are in accordance with the period of stabilization observed in the *mdx* mouse following the “crisis” period that occurs between 3 and 8 weeks of age (76).

Together these analyses have identified disease progression-associated changes in activation state for canonical pathways and upstream regulators in dystrophic muscle. Findings from the pooled analysis (Fig. 4) were generally also consistent in the analyses performed separately at each age, despite lower statistical power in the latter (supplemental Figs. S10, S11, S12).

##### CAV3, MVP, and PAK1 Are Upregulated In Dystrophic Muscle

Three proteins were selected for further validation of the proteomics data: (1) caveolin-3 (CAV3), a consistently elevated sarcolemmal protein, (2) major vault protein (MVP), one of 8 proteins with the highest significance in the global ANOVA analysis comparing all nine groups (adjusted *P* = 6 × 10^−11^) and (3) the serine/threonine-protein kinase, PAK1, which was one of the most highly upregulated proteins in the dystrophic group at all ages (>3-fold in 8-week *mdx*). These proteins were analyzed by Western blot and compared with expression levels in the HiRIEF-LC–MS/MS data (Fig. 7*A*, 7*B*). Similar results were obtained in three independent blots (one representative blot is shown for each protein, Fig. 7*B*). Expression patterns were comparable between HiRIEF-LC–MS/MS and Western blot experiments (Fig. 7*B*), thereby validating the MS data using an orthogonal protein quantification methodology. The expression of the corresponding mRNA transcripts was determined in parallel, which mirrored the protein expression data in the case of *Mvp* and *Pak1*, consistent with their transcriptional upregulation in dystrophic muscle, although some changes between WT and the dystrophic groups did not reach statistical significance at the *P* < 0.05 level (Fig. 7*C*). In contrast, *Cav3* mRNA expression was not significantly increased in *mdx* and *mdx52* samples compared with the WT at 8 and 80 weeks of age.

##### MVP is Upregulated During, but Dispensable for, Myogenic Differentiation

We next sought to further investigate the function of MVP in the commonly used C2C12 murine myoblast cell line. C2C12 cells can be propagated as undifferentiated myoblasts, but upon several days culture in low serum differentiation medium (DM) these cells fuze into syncytial myotubes (Fig. 8*A*). *Mvp* mRNA and MVP protein were expressed at low levels in proliferating myoblasts and progressively upregulated during myogenic differentiation (Fig. 8*B*, 8C). However, depletion of MVP expression by RNAi did not affect myogenic differentiation (Fig. 8*D*, 8*E*) despite high levels of knockdown (Fig. 8*F*, 8*G*). These data suggest that MVP may play a role in, but is dispensable for, myogenic differentiation/muscle regeneration.

## DISCUSSION

The study of the proteomic alterations that underlie diseases such as DMD remains important for understanding disease pathology, identifying novel therapeutic targets, and discovering biomarkers of disease progression or response to therapy. In this study, we have used MS-based profiling to characterize the proteomic signature of dystrophic muscle in two different mouse models of DMD, and at different stages of the disease. Despite the technical challenges associated with proteomics analysis in skeletal muscle, we report quantification of 4974 proteins across all nine conditions in triplicate (*i.e.* WT, *mdx*, and *mdx52* at 8 weeks, 16 weeks, and 80 weeks of age) (Fig. 1), the highest number of quantified proteins in dystrophic muscle described to date.

We selected the TA for proteomic analysis for several reasons, (1) we have previously generated a wealth of comparable proteomics/transcriptomics/miRNomics data in this muscle (39, 61), (2) the TA is accessible for intramuscular injection of therapeutics and toxicants allowing for comparison with data derived from such studies, and (3) experimental restoration of dystrophin restoration in this muscle is relatively straight forward (56, 57, 61, 77). The diaphragm muscle of the *mdx* mouse exhibits more severe degeneration (78) and so high-resolution analyses in this tissue may reveal additional insights into dystrophic pathology. Others have performed proteomics analyses in dystrophic diaphragm, although at lower depth than described here (72, 79).

Initial analyses aimed to identify proteomic changes that might explain the differences in muscle regeneration phenotype observed in the TA when comparing between the *mdx* and *mdx52* strains at 8 and 16 weeks of age (32). However, our data suggest that the skeletal muscle proteomes of these two dystrophic mouse models are very highly similar (Figs. 2, supplemental Fig. S6, S7). Importantly, much of the proteome remains invisible on account of the peptide masking effect (49), failure to consistently detect low abundance protein-derived peptides in all samples, or peptides from some proteins falling outside the narrow pI range used for prefractionation. Based on an update of our previous calculations of protein-coding transcript detection (39), we estimate that ∼35% of the proteome was detected in the analyses described here. As such, putative proteins that are differentially expressed between *mdx52* and *mdx* mice may be invisible to our analysis for reasons independent of statistical power. Alternatively, expression changes in specific subpopulations of cells (*e.g.* infiltrating immune cells) or subsets of myofibers (*e.g.* regenerating fibers) may not be detectable in the context of unchanged protein expression in the bulk population. It is therefore possible that the differences between the *mdx* and *mdx52* models may be explained by expression changes in proteins that could not be detected using this methodology. Future proteomic studies should aim to understand dystrophy-associated expression changes in distinct cell/fiber subtypes.

The pooling of expression data from the *mdx* and *mdx52* strains allowed us to identify key proteins associated with dystrophic pathology, irrespective of mutation (Fig. 3, 4). Pathway analysis was used in an effort to understand the biological relevance of differential protein expression. Mitochondrial disruption was identified by both IPA and gene list enrichment across multiple analyses (Figs. 3, 4, supplemental Fig. S10, and Fig. 6*C* Clusters 2 and 3). A reduction in the number of mitochondria in dystrophic muscle is a possible explanation for these findings and is further supported by the downregulation of the protein components of all five electron transport complexes. Furthermore, the analysis of protein expression across ages suggests that this perturbation of mitochondrial biology worsens with pathological progression (Fig. 3, 6*C*). Nevertheless, Mitochondrial Dysfunction was still observed in the 8 week-old dystrophic samples (Fig. 3, S10). Percival *et al.* reported a reduction in the density of the subsarcolemmal mitochondria pool in *mdx* TA muscle (80), and *mdx*-derived myoblasts contain fewer mitochondria than their WT counterparts (81), consistent with this hypothesis.

Upstream regulator analysis identified multiple master regulators of mitochondrial biogenesis as being perturbed in dystrophic muscle. These included the peroxisome proliferator activated receptor family members: PPARA, PPARG, and PPARD, their associated transcriptional co-activators: PPARGC1A (PGC-1α), and PPARGC1B (PGC-1β), and the mitochondria-specific transcription factor TFAM (Fig. 4, supplemental Fig. S10*C*, S12). (Notably, downregulation of PGC-1α protein itself has been reported in the vastus lateralis muscle of the dystrophic GRMD dog (46)). Overlap between differentially expressed proteins enriched in PPAR, PGC-1α, PGC-1β, and TFAM was minimal (data not shown), suggesting *bona fide* perturbation of these distinct regulators as opposed to enrichment because of high numbers of differentially expressed proteins that are common between gene lists. Downregulation of PGC-1α activity, and the resulting loss in mitochondrial numbers/function, may therefore account for the observed inhibition of the canonical pathways associated with mitochondrial dysfunction, oxidative phosphorylation, fatty acid oxidation, and gluconeogenesis (82) (Fig. 4). Multiple factors may contribute to downregulation of PGC-1α. For example, AMPK (5′ AMP-activated protein kinase) and SIRT1 activate PGC-1α by phosphorylation and deacetylation respectively (83), both of which were predicted to be downregulated by upstream regulator analysis (Fig. 4).

Several inflammation-associated canonical pathways and upstream regulators were predicted to be upregulated, consistent with the well-described persistent inflammation that accompanies myonecrosis and regeneration in dystrophic muscle (84). These included tumor necrosis factor (TNF), interferon gamma (INFG, INF-γ) (85) and the pro-fibrotic transforming growth factor beta (TGFB1, TGF-β) (Fig. 4). Pro-inflammatory cytokines are likely derived from the infiltrating immune cells. However, TNF and INFG activate the NF-κB (Nuclear factor kappa-light-chain-enhancer of activated B cells), a transcription factor that acts as a master regulator of inflammation, which in turn results in feed-forward activation of TNF and IFNG expression in the myofibers themselves. Activation of NF-κB is a well-described feature of dystrophic muscle (13, 86, 87) but was not identified as being upregulated in our data set, possibly because of limited proteome coverage. Conversely, the NF-κB inhibitor (NFKBIA, IκBα) was paradoxically predicted to be upregulated. This is consistent with the reported auto-regulatory feedback loop by which NF-κB promotes the expression of its inhibitor (88). Interestingly, NF-κB negatively regulates PGC-1α via a direct binding interaction (89, 90), and TNF has been shown to also suppress PGC-1α in a cardiac cell model (91), suggestive of a link between inflammatory and metabolic pathologies in dystrophic muscle.

PI3K/AKT Signaling was the most highly activated canonical pathway in dystrophic muscle, consistent with previous observations in the *mdx* diaphragm (92). Activation of PI3K/AKT can result in muscle hypertrophy (93), suggesting that this may explain the increase in fiber size observed in *mdx* muscle. Similarly, AKT-mediated inactivation of FOXO transcription factors leads to the inhibition of atrophic signals, which may explain the reduced expression of MSTN in dystrophic muscle. (FOXO3 signaling was predicted to be downregulated based the data reported here).

Insulin receptor signaling was found to be the most strongly downregulated upstream regulator, suggesting that insulin signaling is not causative of PI3K/AKT pathway activation in this context. Alternative possibilities include Integrin Signaling, ILK (integrin-linked kinase), and RICTOR (a subunit of mTOR complex 2 (mTORC2)) that were all upregulated in dystrophic muscle (Figs. 4, supplemental Figs. S^10^, S^11^, S^12^) and are possible factors contributing to PI3K/AKT activation (94, 95). ILK signaling was also identified by pathway analysis in our previous study in *mdx* TA (39), and others have similarly reported ILK activation in the *mdx* tissues (47, 92). Additionally, there is some evidence that inflammatory cytokines such as TNF and IFNG can stimulate PI3K/AKT (96).

PI3K/AKT itself contributes to the activation of the NF-κB via the phosphorylation of IKK, which in turn inhibits IκBα by phosphorylation (92) and has been shown to directly inhibit PGC-1α via phosphorylation in liver cells (97). Taken together, these data point to interactions between metabolic, mitochondrial biogenesis, inflammatory, and muscle growth pathways in dystrophic muscle (Fig. 9).

Notably, many of the regulatory proteins identified above have been explored as potential targets for pharmacological manipulation (Fig. 9). For example, PGC-1α gain-of-function and pharmacological activation of PPARD have been shown to be beneficial in the *mdx* mouse (98), and reverse mitochondrial defects in *mdx* myoblasts (81), respectively. Activation of AMPK and SIRT1 (by AICAR (99) and resveratrol (100) respectively) has been shown to attenuate pathology in the *mdx* mouse, also a likely consequence of PGC-1α upregulation. Treatment with NF-κB inhibitors (such as CAT-1041 and edasalonexent) ameliorated dystrophic pathology in *mdx* mice and GRMD dogs (101) and has shown promise in a phase 1 clinical trial in DMD patients (102). Treatment with the anti-TNF antibody infliximab (brand name: Remicade) improved dystrophic pathology by reducing myonecrosis (103), and cardiac fibrosis (104) in dystrophic mice, although detrimental changes in heart function were observed attributable to inhibition of the PI3K/AKT pathway (104).

Pathway analysis with IPA is appealing as it takes expression fold changes into account, generates Z-score outputs for pathway activation state that are easily interpretable, allows for exploration of regulatory relationships, and includes the prediction of perturbed upstream regulators. However, any pathway analysis is necessarily dependent on the quality of its gene list annotations. As such, we have found that IPA frequently does not identify muscle associated pathways/regulators (data not shown). In contrast, multiple muscle-associated GO terms were identified using a less sophisticated gene list enrichment approach (55). This was particularly apparent in the list of the most differentially expressed proteins (Fig. 5*A*). We have therefore performed both types of analysis in this study.

A recent study by Capitanio *et al.* described a label free MS-based proteomics analysis of vastus lateralis muscles biopsied from DMD and BMD patients, compared with healthy controls (*n* = 3) (48). To determine whether the findings reported here could be recapitulated in human muscle we compared all proteins found to be differentially expressed in DMD or BMD relative to controls against all proteins found to be significantly altered in any of the dystrophic *versus* WT comparisons described in this study. 135 proteins were commonly identified between DMD/BMD patients and dystrophic mice, thereby providing independent evidence that many of the findings reported here are relevant to the situation in dystrophic patient muscle (supplemental Fig. S13*A*). Indeed, this level of overlap is perhaps surprising when considering the differences in organism, muscle type, mutation type, disease-stage progression, and the methodologies used. Conversely, 91 proteins uniquely identified in the human study, of these 48 were also detected in our datasets, but were not differentially expressed, with the remaining 43 proteins invisible in our analyses (supplemental Fig. S13*B*). 2458 proteins were uniquely identified as being differentially expressed in the dystrophic mouse samples, which emphasizes the benefit of the high-resolution proteomics approach used in this study, in terms of deep proteome coverage. Differentially expressed proteins that were common to dystrophic human and mouse muscle were enriched for GO terms associated with muscle structure, the cytoskeleton, and energy production (supplemental Fig. S13*C*) consistent with findings described above (Fig. 3). Proteins that were uniquely differentially expressed in the human patient data set were enriched for antioxidant activity, peroxidase activity, exocytosis, and vesicle structure and function (supplemental Fig. S13*D*). Notably the *P*-values for these enriched GO terms were relatively low. Proteins that were uniquely differentially expressed in our murine datasets were enriched for RNA binding, multiple metabolic processes, and the mitochondrion (supplemental Fig. *S13E*) similar to findings described above (Fig. 3). *P*-values for the enrichment of these GO terms were comparatively very high, which is to be expected when considering the greater number of proteins available for analysis.

A possible limitation of this study is the influence of batch effects, as experimental samples for each age were split between three separate pools of TMT-labeled peptides. This study design ensured that statistical tests performed within each age are not influenced by batch effects. However, comparisons between ages may be subject to this technical source of variation, although there is evidence that such effects are minimal in our data. Firstly, the normalization of TMT ratios to the bridge sample minimizes batch effects to some extent, and the proteomics performance for the bridge sample was highly similar for each TMT10 set (supplemental Fig. S3). Secondly, comparing TMT ratios across all samples for known proteins that are differentially expressed in dystrophic muscle shows minimal differences between ages/TMT10 sets (supplemental Fig. S5), and the majority of proteins are not changed over time (Fig. 3, 4, supplemental Fig. S^6^, File S^1^). Thirdly, proteins/pathways that are altered with age are consistent with established features of dystrophic pathology (such as the accumulation of fibrotic damage) (Fig. 6, supplemental Fig. S10, S11, S12).

This study has highlighted multiple proteins of interest, of which we selected MVP for further investigation. MVP is the major constituent of vault particles, ribonucleoprotein structures (the function of which is poorly understood) (105). MVP was upregulated during, but dispensable for, myogenic differentiation (Figs. 7, 8) suggesting that MVP upregulation may be associated with muscle regeneration in dystrophic muscle. Alternatively, MVP may reflect a different facet of dystrophic pathology, as this protein has been shown to be elevated in response to serum-starvation-induced apoptotic stress (106). Notably, the other protein components of vault particles (*i.e.* TEP1 and PARP4) were similarly upregulated in dystrophic muscle, although to a lesser extent, suggesting an increase in the number of vaults may explain these data. Recent evidence has shown that vault particles can act as scaffolds and are involved in cell survival signaling (107, 108). Moreover, they have been reported to regulate growth and survival of respiratory smooth muscle cells (108). To our knowledge, this is the first study demonstrating an association between MVP/vault particles and dystrophic pathology, which is deserving of further investigation.

Using two dystrophic mouse models and the inclusion of different disease time points, this study provides a wider perspective of, and offers new insights into, the pathological mechanisms involved in Duchenne muscular dystrophy. The data presented here describe a wealth of novel differentially expressed proteins, perturbed biological pathways, and predicted upstream regulators. This data set will constitute a valuable resource for the DMD research community, and for those studying the effects of aging on the WT muscle proteome.

## DATA AVAILABILITY

Protein expression data and statistical analysis are summarized in File S1. All other data relating to this manuscript are freely available on request from the authors. The MS raw proteomics data have been deposited to the ProteomeXchange Consortium via the JPOST partner repository with the data set identifier PXD017169 (JPST000734).

## Supplementary Material

supplemental Fig. S4

File S1

Supplemental Data

## References

[1] Moriuchi T., Kagawa N., Mukoyama M., and Hizawa K. (1993) Autopsy analyses of the muscular dystrophies. Tokushima J. Exp. Med. 40, 83–93 8211986

[2] Chiang D. Y., Allen H. D., Kim J. J., Valdes S. O., Wang Y., Pignatelli R. H., Lotze T. E., and Miyake C. Y. (2016) Relation of cardiac dysfunction to rhythm abnormalities in patients with Duchenne or Becker muscular dystrophies. Am. J. Cardiol. 117, 1349–1354 26952271 10.1016/j.amjcard.2016.01.031

[3] Ishikawa Y. Y., Miura T., Ishikawa Y. Y., Aoyagi T., Ogata H., Hamada S., and Minami R. (2011) Duchenne muscular dystrophy: Survival by cardio-respiratory interventions. Neuromuscul. Disord. 21, 47–51 21144751 10.1016/j.nmd.2010.09.006

[4] Ricotti V., Ridout D. A., Scott E., Quinlivan R., Robb S. A., Manzur A. Y., Muntoni F., Manzur A., Muntoni F., Robb S., Quinlivan R., Ricotti V., Main M., Bushby K., Straub V., Sarkozy A., Guglieri M., Strehle E., Eagle M., Mayhew A., Roper H., McMurchie H., Childs A., Pysden K., Pallant L., Spinty S., Peachey G., Shillington A., Wraige E., Jungbluth H., Sheehan J., Spahr R., Hughes I., Bateman E., Cammiss C., Willis T., Groves L., Emery N., Baxter P., Senior M., Hartley L., Parsons B., Majumdar A., Jenkins L., Naismith K., Keddie A., Horrocks I., Di Marco M., Chow G., Miah A., de Goede C., Thomas N., Geary M., Palmer J., White C., Greenfield K., and Scott E, on behalf of the NorthStar Clinical Network, (2013) Long-term benefits and adverse effects of intermittent versus daily glucocorticoids in boys with Duchenne muscular dystrophy. J. Neurol. Neurosurg. Psychiatry 84, 698–705 23250964 10.1136/jnnp-2012-303902

[5] Aartsma-Rus A., Van Deutekom J. C. T., Fokkema I. F., Van Ommen G. J. B., and Den Dunnen J. T. (2006) Entries in the Leiden Duchenne muscular dystrophy mutation database: An overview of mutation types and paradoxical cases that confirm the reading-frame rule. Muscle Nerve. 34, 135–144 16770791 10.1002/mus.20586

[6] Bladen C. L., Salgado D., Monges S., Foncuberta M. E., Kekou K., Kosma K., Dawkins H., Lamont L., Roy A. J., Chamova T., Guergueltcheva V., Chan S., Korngut L., Campbell C., Dai Y., Wang J., Barišić N., Brabec P., Lahdetie J., Walter M. C., Schreiber-Katz O., Karcagi V., Garami M., Viswanathan V., Bayat F., Buccella F., Kimura E., Koeks Z., van den Bergen J. C., Rodrigues M., Roxburgh R., Lusakowska A., Kostera-Pruszczyk A., Zimowski J., Santos R., Neagu E., Artemieva S., Rasic V. M., Vojinovic D., Posada M., Bloetzer C., Jeannet P.-Y., Joncourt F., Díaz-Manera J., Gallardo E., Karaduman A. A., Topaloğlu H., El Sherif R., Stringer A., Shatillo A. V., Martin A. S., Peay H. L., Bellgard M. I., Kirschner J., Flanigan K. M., Straub V., Bushby K., Verschuuren J., Aartsma-Rus A., Béroud C., and Lochmüller H. (2015) The TREAT-NMD DMD global database: Analysis of more than 7,000 duchenne muscular dystrophy mutations. Hum. Mutat. 36, 395–402 25604253 10.1002/humu.22758 PMC4405042

[7] Hoffman E. P., Brown R. H., and Kunkel L. M. (1987) Dystrophin: the protein product of the Duchene muscular dystrophy locus. 1987. Cell 51, 919–928 3319190 10.1016/0092-8674(87)90579-4

[8] Levine B. A., Moir A. J. G., Patchell V. B., and Perry S. V. (1990) The interaction of actin with dystrophin. FEBS Lett. 263, 159–162 2185033 10.1016/0014-5793(90)80728-2

[9] Samitt C. E., and Bonilla E. (1990) Immunocytochemical study of dystrophin at the myotendinous junction. Muscle Nerve. 13, 493–500 2195339 10.1002/mus.880130605

[10] Muntoni F., Torelli S., and Ferlini A. (2003) Dystrophin and mutations: One gene, several proteins, multiple phenotypes. Lancet. Neurol. 2, 731–740 14636778 10.1016/s1474-4422(03)00585-4

[11] van Westering T. L. E., Betts C. A., and Wood M. J. A. (2015) Current understanding of molecular pathology and treatment of cardiomyopathy in duchenne muscular dystrophy. Molecules 20, 8823–8855 25988613 10.3390/molecules20058823 PMC6272314

[12] Pastoret C., and Sebille A. (1995) mdx mice show progressive weakness and muscle deterioration with age. J. Neurol. Sci. 129, 97–105 7608742 10.1016/0022-510x(94)00276-t

[13] Messina S., Bitto A., Aguennouz M., Minutoli L., Monici M. C., Altavilla D., Squadrito F., and Vita G. (2006) Nuclear factor kappa-B blockade reduces skeletal muscle degeneration and enhances muscle function in Mdx mice. Exp. Neurol. 198, 234–241 16410003 10.1016/j.expneurol.2005.11.021

[14] Yokota T., Lu Q.-L., Morgan J. E., Davies K. E., Fisher R., Takeda S., and Partridge T. A. (2006) Expansion of revertant fibers in dystrophic mdx muscles reflects activity of muscle precursor cells and serves as an index of muscle regeneration. J. Cell Sci. 119, 2679–2687 16757519 10.1242/jcs.03000

[15] Doorenweerd N., Mahfouz A., van Putten M., Kaliyaperumal R., T' Hoen P. A. C., Hendriksen J. G. M., Aartsma-Rus A. M., Verschuuren J. J. G. M., Niks E. H., Reinders M. J. T., Kan H. E., and Lelieveldt B. P. F. (2017) Timing and localization of human dystrophin isoform expression provide insights into the cognitive phenotype of Duchenne muscular dystrophy. Sci. Rep 7, 12575 28974727 10.1038/s41598-017-12981-5 PMC5626779

[16] Austin R. C., Howard P. L., D'Souza V. N., Klamut H. J., and Ray P. N. (1995) Cloning and characterization of alternatively spliced isoforms of Dp71. Hum. Mol. Genet. 4, 1475–1483 8541829 10.1093/hmg/4.9.1475

[17] D'Souza V. N., Nguyen T. M., Morris G. E., Karges W., Pillers D. A., and Ray P. N. (1995) A novel dystrophin isoform is required for normal retinal electrophysiology. Hum. Mol. Genet. 4, 837–842 7633443 10.1093/hmg/4.5.837

[18] Daoud F., Angeard N., Demerre B., Martie I., Benyaou R., Leturcq F., Cossée M., Deburgrave N., Saillour Y., Tuffery S., Urtizberea A., Toutain A., Echenne B., Frischman M., Mayer M., Desguerre I., Estournet B., Réveillère C., P.-B., Cuisset J. M., Kaplan J. C., Héron D., Rivier F., and Chelly J. (2009) Analysis of Dp71 contribution in the severity of mental retardation through comparison of Duchenne and Becker patients differing by mutation consequences on Dp71 expression. Hum. Mol. Genet. 18, 3779–3794 19602481 10.1093/hmg/ddp320

[19] Moizard M. P., Toutain A., Fournier D., Berret F., Raynaud M., Billard C., Andres C., and Moraine C. (2000) Severe cognitive impairment in DMD: obvious clinical indication for Dp71 isoform point mutation screening. Eur. J. Hum. Genet. EJHG 8, 552–556 10909857 10.1038/sj.ejhg.5200488

[20] McGreevy J. W., Hakim C. H., McIntosh M. A., and Duan D. (2015) Animal models of Duchenne muscular dystrophy: from basic mechanisms to gene therapy. Dis. Model. Mech. 8, 195–213 25740330 10.1242/dmm.018424 PMC4348559

[21] Bulfield G., Siller W. G., Wight P. A., and Moore K. J. (1984) X chromosome-linked muscular dystrophy (mdx) in the mouse. Proc. Natl. Acad. Sci. U S A 81, 1189–1192 6583703 10.1073/pnas.81.4.1189 PMC344791

[22] Sicinski P., Geng Y., Ryder-Cook A. S., Barnard E. A., Darlison M. G., and Barnard P. J. (1989) The molecular basis of muscular dystrophy in the mdx mouse: a point mutation. Science 244, 1578–1580 2662404 10.1126/science.2662404

[23] Duddy W., Duguez S., Johnston H., Cohen T. V., Phadke A., Gordish-Dressman H., Nagaraju K., Gnocchi V., Low S., and Partridge T. (2015) Muscular dystrophy in the mdx mouse is a severe myopathy compounded by hypotrophy, hypertrophy and hyperplasia. Skelet. Muscle. 5, 16 25987977 10.1186/s13395-015-0041-y PMC4434871

[24] Chamberlain J. S., Metzger J., Reyes M., Townsend D., and Faulkner J. A. (2007) Dystrophin-deficient mdx mice display a reduced life span and are susceptible to spontaneous rhabdomyosarcoma. FASEB J. Off 21, 2195–2204 10.1096/fj.06-7353com 17360850

[25] Dangain J., and Vrbova G. (1984) Muscle development in mdx mutant mice. Muscle Nerve. 7, 700–704 6543918 10.1002/mus.880070903

[26] Turk R., Sterrenburg E., de Meijer E. J., van Ommen G.-J. B., den Dunnen J. T., and 't Hoen P. A. C. (2005) Muscle regeneration in dystrophin-deficient mdx mice studied by gene expression profiling. BMC Genomics 6, 98 16011810 10.1186/1471-2164-6-98 PMC1190170

[27] Roig M., Roma J., Fargas A., and Munell F. (2004) Longitudinal pathologic study of the gastrocnemius muscle group in mdx mice. Acta Neuropathol.) 107, 27–34 14530991 10.1007/s00401-003-0773-3

[28] Lefaucheur J. P., Pastoret C., and Sebille A. (1995) Phenotype of dystrophinopathy in old mdx mice. Anat. Rec. 242, 70–76 7604983 10.1002/ar.1092420109

[29] Stuckey D. J., Carr C. A., Camelliti P., Tyler D. J., Davies K. E., and Clarke K. (2012) In vivo MRI characterization of progressive cardiac dysfunction in the mdx mouse model of muscular dystrophy. PLoS ONE. 7, e28569 22235247 10.1371/journal.pone.0028569 PMC3250389

[30] The UMD-TREAT-NMD DMD Locus Specific Databases The UMD TREAT-NMD DMD mutations database

[31] Araki E., Nakamurab K., Nakaob K., Kameyac S., Kobayashid O., Nonakad I., Kobayashia T., Katsuki M., Araki E., Nakamura K., Nakao K., Kameya S., Kobayashi O., Nonaka I., and Kobayashi T. (1997) Targeted disruption of exon 52 in the mouse dystrophin gene induced muscle degeneration similar to that observed in Duchenne muscular dystrophy. Biochem. Biophys. Res. Commun. 238, 492–497 9299538 10.1006/bbrc.1997.7328

[32] Echigoya Y., Lee J., Rodrigues M., Nagata T., Tanihata J., Nozohourmehrabad A., Panesar D., Miskew B., Aoki Y., and Yokota T. (2013) Mutation types and aging differently affect revertant fiber expansion in dystrophic mdx and mdx52 mice. PLoS ONE. 8, e69194 23894429 10.1371/journal.pone.0069194 PMC3722172

[33] Echigoya Y., Aoki Y., Miskew B., Panesar D., Touznik A., Nagata T., Tanihata J., Nakamura A., Nagaraju K., and Yokota T. (2015) Long-term efficacy of systemic multiexon skipping targeting Dystrophin exons 45-55 with a cocktail of vivo-morpholinos in Mdx52 mice. Mol. Ther. - Nucleic Acids 4, e225 25647512 10.1038/mtna.2014.76 PMC4345310

[34] Echigoya Y., Lim K. R. Q., Trieu N., Bao B., Miskew Nichols B., Vila M. C., Novak J. S., Hara Y., Lee J., Touznik A., Mamchaoui K., Aoki Y., Takeda S., Nagaraju K., Mouly V., Maruyama R., Duddy W., and Yokota T. (2017) Quantitative antisense screening and optimization for exon 51 skipping in Duchenne muscular dystrophy. Mol. Ther. J. 25, 2561–2572 10.1016/j.ymthe.2017.07.014 PMC5675502 28865998

[35] Miyatake S., Mizobe Y., Takizawa H., Hara Y., Yokota T., Takeda S., and Aoki Y. (2018) Exon skipping therapy using phosphorodiamidate morpholino oligomers in the mdx52 mouse model of Duchenne muscular dystrophy. Methods Mol. Biol. Clifton NJ 1687, 123–141 10.1007/978-1-4939-7374-3_9 29067660

[36] Haslett J. N., Sanoudou D., Kho A. T., Bennett R. R., Greenberg S. A., Kohane I. S., Beggs A. H., and Kunkel L. M. (2002) Gene expression comparison of biopsies from Duchenne muscular dystrophy (DMD) and normal skeletal muscle. Proc. Natl. Acad. Sci. U S A 99, 15000–15005 12415109 10.1073/pnas.192571199 PMC137534

[37] Tian L. J., Cao J. H., Deng X. Q., Zhang C. L., Qian T., Song X. X., and Huang B. S. (2014) Gene expression profiling of Duchenne muscular dystrophy reveals characteristics along disease progression. Genet. Mol. Res. 13, 1402–1411 24634239 10.4238/2014.February.28.13

[38] Brinkmeyer-Langford C., Chu C., Balog-Alvarez C., Yu X., Cai J. J., Nabity M., and Kornegay J. N. (2018) Expression profiling of disease progression in canine model of Duchenne muscular dystrophy. PLoS ONE. 13, e0194485 29554127 10.1371/journal.pone.0194485 PMC5858769

[39] Roberts T. C., Johansson H. J., McClorey G., Godfrey C., Blomberg K. E. M., Coursindel T., Gait M. J., Smith C. I. E., Lehtiö J., EL Andaloussi S., and Wood M. J. A. (2015) Multi-level omics analysis in a murine model of dystrophin loss and therapeutic restoration. Hum. Mol. Genet. 24, 6756–6758 26385637 10.1093/hmg/ddv381 PMC4634378

[40] 't Hoen P. A. C., van der Wees C. G. C., Aartsma-Rus A., Turk R., Goyenvalle A., Danos O., Garcia L., van Ommen G.-J. B., den Dunnen J. T., and van Deutekom J. C. T. (2006) Gene expression profiling to monitor therapeutic and adverse effects of antisense therapies for Duchenne muscular dystrophy. Pharmacogenomics 7, 281–297 16610940 10.2217/14622416.7.3.281

[41] Gygi S. P., Rochon Y., Franza B. R., and Aebersold R. (1999) Correlation between protein and mRNA abundance in yeast. Mol. Cell Biol. 19, 1720–1730 10022859 10.1128/mcb.19.3.1720 PMC83965

[42] Nie L., Wu G., and Zhang W. (2006) Correlation between mRNA and protein abundance in Desulfovibrio vulgaris: A multiple regression to identify sources of variations. Biochem. Biophys. Res. Commun. 339, 603–610 16310166 10.1016/j.bbrc.2005.11.055

[43] Marotta M., Ruiz-Roig C., Sarria Y., Peiro J. L., Nuñez F., Ceron J., Munell F., and Roig-Quilis M. (2009) Muscle genome-wide expression profiling during disease evolution in mdx mice. Physiol. Genomics. 37, 119–132 19223608 10.1152/physiolgenomics.90370.2008

[44] Gardan-Salmon D., Dixon J. M., Lonergan S. M., and Selsby J. T. (2011) Proteomic assessment of the acute phase of dystrophin deficiency in mdx mice. Eur. J. Appl. Physiol. 111, 2763–2773 21409400 10.1007/s00421-011-1906-3

[45] Carberry S., Zweyer M., Swandulla D., and Ohlendieck K. (2012) Profiling of age-related changes in the tibialis anterior muscle proteome of the mdx mouse model of dystrophinopathy. J. Biomed. Biotechnol. 2012, 1–11 23093855 10.1155/2012/691641 PMC3471022

[46] Guevel L., Lavoie J. R., Perez-Iratxeta C., Rouger K., Dubreil L., Feron M., Talon S., Brand M., and Megeney L. A. (2011) Quantitative proteomic analysis of dystrophic dog muscle. J. Proteome Res. 10, 2465–2478 21410286 10.1021/pr2001385

[47] Rayavarapu S., Coley W., Cakir E., Jahnke V., Takeda S., Aoki Y., Grodish-Dressman H., Jaiswal J. K., Hoffman E. P., Brown K. J., Hathout Y., and Nagaraju K. (2013) Identification of disease specific pathways using in vivo SILAC proteomics in dystrophin deficient mdx mouse. Mol. Cell. Proteomics 12, 1061–1073 23297347 10.1074/mcp.M112.023127 PMC3650321

[48] Capitanio D., Moriggi M., Torretta E., Barbacini P., De Palma S., Viganò A., Lochmüller H., Muntoni F., Ferlini A., Mora M., and Gelfi C. (2020) Comparative proteomic analyses of Duchenne muscular dystrophy and Becker muscular dystrophy muscles: changes contributing to preserve muscle function in Becker muscular dystrophy patients. J. Cachexia. Sarcopenia Muscle. 11, 547–563 31991054 10.1002/jcsm.12527 PMC7113522

[49] Ohlendieck K. (2011) Skeletal muscle proteomics: current approaches, technical challenges and emerging techniques. Skelet. Muscle 1, 6 21798084 10.1186/2044-5040-1-6 PMC3143904

[50] Murphy S., Zweyer M., Raucamp M., Henry M., Meleady P., Swandulla D., and Ohlendieck K. (2019) Proteomic profiling of the mouse diaphragm and refined mass spectrometric analysis of the dystrophic phenotype. J. Muscle Res. Cell Motil. 40, 9–28 30888583 10.1007/s10974-019-09507-z

[51] Roberts T. C., Coenen-Stass A. M. L., Betts C. A., and Wood M. J. A. (2014) Detection and quantification of extracellular microRNAs in murine biofluids. Biol. Proced. Online 16, 5 24629058 10.1186/1480-9222-16-5 PMC3995583

[52] Roberts T. C., Coenen-Stass A. M. L., and Wood M. J. A. (2014) Assessment of RT-qPCR normalization strategies for accurate quantification of extracellular microRNAs in murine Serum. PLoS ONE. 9, e89237 24586621 10.1371/journal.pone.0089237 PMC3929707

[53] Branca R. M. M., Orre L. M., Johansson H. J., Granholm V., Huss M., Pérez-Bercoff A., Forshed J., Käll L., and Lehtiö J. (2014) HiRIEF LC-MS enables deep proteome coverage and unbiased proteogenomics. Nat. Methods 11, 59–62 24240322 10.1038/nmeth.2732

[54] Saeed A. I., Sharov V., White J., Li J., Liang W., Bhagabati N., Braisted J., Klapa M., Currier T., Thiagarajan M., Sturn A., Snuffin M., Rezantsev A., Popov D., Ryltsov A., Kostukovich E., Borisovsky I., Liu Z., Vinsavich A., Trush V., and Quackenbush J. (2003) TM4: a free, open-source system for microarray data management and analysis. BioTechniques 34, 374–378 12613259 10.2144/03342mt01

[55] Chen J., Bardes E. E., Aronow B. J., and Jegga A. G. (2009) ToppGene Suite for gene list enrichment analysis and candidate gene prioritization. Nucleic Acids Res. 37, W305–W311 19465376 10.1093/nar/gkp427 PMC2703978

[56] Roberts T. C., Blomberg K. E. M., McClorey G., El Andaloussi S., Godfrey C., Betts C., Coursindel T., Gait M. J., Smith C. E. I. E., and Wood M. J. A. (2012) Expression analysis in multiple muscle groups and serum reveals complexity in the MicroRNA transcriptome of the mdx mouse with implications for therapy. Mol. Ther. Nucleic Acids 1, e39 23344181 10.1038/mtna.2012.26 PMC3437806

[57] Roberts T. C., Godfrey C., McClorey G., Vader P., Briggs D., Gardiner C., Aoki Y., Sargent I., Morgan J. E., and Wood M. J. A. (2013) Extracellular microRNAs are dynamic non-vesicular biomarkers of muscle turnover. Nucleic Acids Res. 41, 9500–9513 23945935 10.1093/nar/gkt724 PMC3814379

[58] Coenen-Stass A. M. L., Betts C. A., Lee Y. F., Mäger I., Turunen M. P., El Andaloussi S., Morgan J. E., Wood M. J. A., and Roberts T. C. (2016) Selective release of muscle-specific, extracellular microRNAs during myogenic differentiation. Hum. Mol. Genet. 25, 3960–3974 27466195 10.1093/hmg/ddw237 PMC5291232

[59] Mizuno H., Nakamura A., Aoki Y., Ito N., Kishi S., Yamamoto K., Sekiguchi M., Takeda S., and Hashido K. (2011) Identification of muscle-specific MicroRNAs in serum of muscular dystrophy animal models: Promising novel blood-based markers for muscular dystrophy. PLoS ONE. 6, e18388 21479190 10.1371/journal.pone.0018388 PMC3068182

[60] Cacchiarelli D., Legnini I., Martone J., Cazzella V., D'Amico A., Bertini E., and Bozzoni I. (2011) miRNAs as serum biomarkers for Duchenne muscular dystrophy. EMBO Mol. Med 3, 258–265 21425469 10.1002/emmm.201100133 PMC3112257

[61] Coenen-Stass A. M. L., Sork H., Gatto S., Godfrey C., Bhomra A., Krjutškov K., Hart J. R., Westholm J. O., O'Donovan L., Roos A., Lochmüller H., Puri P. L., El Andaloussi S., Wood M. J. A., and Roberts T. C. (2018) Comprehensive RNA-sequencing analysis in serum and muscle reveals novel small RNA signatures with biomarker potential for DMD. Mol. Ther. Nucleic Acids 13, 1–15 30219269 10.1016/j.omtn.2018.08.005 PMC6140421

[62] Coenen-Stass A. M. L., Wood M. J. A., and Roberts T. C. (2017) Biomarker potential of extracellular miRNAs in Duchenne muscular dystrophy. Trends Mol. Med. 23, 989–1001 28988850 10.1016/j.molmed.2017.09.002

[63] Goyenvalle A., Babbs A., Wright J., Wilkins V., Powell D., Garcia L., and Davies K. E. (2012) Rescue of severely affected dystrophin/utrophin-deficient mice through scAAV-U7snRNA-mediated exon skipping. Hum. Mol. Genet. 21, 2559–2571 22388933 10.1093/hmg/dds082 PMC3349427

[64] Ohlendieck K., and Campbell K. P. (1991) Dystrophin-associated proteins are greatly reduced in skeletal muscle from mdx mice. J. Cell Biol. 115, 1685–1694 1757468 10.1083/jcb.115.6.1685 PMC2289197

[65] Ervasti J. M., Ohlendieck K., Kahl S. D., Gaver M. G., and Campbell K. P. (1990) Deficiency of glycoprotein component of the dystrophin complex in dystrophic muscle Deficiency of a glycoprotein component of the dystrophin complex in dystrophic muscle. Nature 345, 315–319 2188135 10.1038/345315a0

[66] Tinsley J., Robinson N., and Davies K. E. (2015) Safety, tolerability, and pharmacokinetics of SMT C1100, a 2-arylbenzoxazole utrophin modulator, following single- and multiple-dose administration to healthy male adult volunteers. J. Clin. Pharmacol. 55, 698–707 25651188 10.1002/jcph.468 PMC5024067

[67] Pan X., Liu J., Nguyen T., Liu C., Sun J., Teng Y., Fergusson M. M., Rovira I. I., Allen M., Springer D. A., Aponte A. M., Gucek M., Balaban R. S., Murphy E., and Finkel T. (2013) The physiological role of mitochondrial calcium revealed by mice lacking the mitochondrial calcium uniporter. Nat. Cell Biol. 15, 1464–1472 24212091 10.1038/ncb2868 PMC3852190

[68] Kleopa K. A., Drousiotou A., Mavrikiou E., Ormiston A., and Kyriakides T. (2006) Naturally occurring utrophin correlates with disease severity in Duchenne muscular dystrophy. Hum. Mol. Genet. 15, 1623–1628 16595608 10.1093/hmg/ddl083

[69] Nguyen T. M., Ellis J. M., Love D. R., Davies K. E., Gatter K. C., Dickson G., and Morris G. E. (1991) Localization of the DMDL gene-encoded dystrophin-related protein using a panel of nineteen monoclonal antibodies: presence at neuromuscular junctions, in the sarcolemma of dystrophic skeletal muscle, in vascular and other smooth muscles, and in proliferating brain cell lines. J. Cell Biol. 115, 1695–1700 1757469 10.1083/jcb.115.6.1695 PMC2289198

[70] Mariot V., Joubert R., Hourdé C., Féasson L., Hanna M., Muntoni F., Maisonobe T., Servais L., Bogni C., Le Panse R., Benvensite O., Stojkovic T., Machado P. M., Voit T., Buj-Bello A., and Dumonceaux J. (2017) Downregulation of myostatin pathway in neuromuscular diseases may explain challenges of anti-myostatin therapeutic approaches. Nat. Commun. 8, 1859 29192144 10.1038/s41467-017-01486-4 PMC5709430

[71] Wakayama Y., Jimi T., Inoue M., Kojima H., Murahashi M., Kumagai T., Yamashita S., Hara H., and Shibuya S. (2002) Reduced aquaporin 4 expression in the muscle plasma membrane of patients with Duchenne muscular dystrophy. Arch. Neurol. 59, 431–437 11890849 10.1001/archneur.59.3.431

[72] Holland A., Dowling P., Meleady P., Henry M., Zweyer M., Mundegar R. R., Swandulla D., and Ohlendieck K. (2015) Label-free mass spectrometric analysis of the mdx-4cv diaphragm identifies the matricellular protein periostin as a potential factor involved in dystrophinopathy-related fibrosis. Proteomics 15, 2318–2331 25737063 10.1002/pmic.201400471

[73] Murphy S., Brinkmeier H., Krautwald M., Henry M., Meleady P., and Ohlendieck K. (2017) Proteomic profiling of the dystrophin complex and membrane fraction from dystrophic mdx muscle reveals decreases in the cytolinker desmoglein and increases in the extracellular matrix stabilizers biglycan and fibronectin. J. Muscle Res. Cell Motil. 38, 251–268 28803268 10.1007/s10974-017-9478-4

[74] Sato S., Omori Y., Katoh K., Kondo M., Kanagawa M., Miyata K., Funabiki K., Koyasu T., Kajimura N., Miyoshi T., Sawai H., Kobayashi K., Tani A., Toda T., Usukura J., Tano Y., Fujikado T., and Furukawa T. (2008) Pikachurin, a dystroglycan ligand, is essential for photoreceptor ribbon synapse formation. Nat. Neurosci. 11, 923–931 18641643 10.1038/nn.2160

[75] Duance V. C., Stephens H. R., Dunn M., Bailey A. J., and Dubowitz V. (1980) A role for collagen in the pathogenesis of muscular dystrophy?. Nature 284, 470–472 7360283 10.1038/284470a0

[76] Tanabe Y., Esaki K., and Nomura T. (1986) Skeletal muscle pathology in X chromosome-linked muscular dystrophy (mdx) mouse. Acta Neuropathol.) 69, 91–95 3962599 10.1007/BF00687043

[77] Betts C., Saleh A. F., Arzumanov A. A., Hammond S. M., Godfrey C., Coursindel T., Gait M. J., and Wood M. J. (2012) Pip6-PMO, a new generation of peptide-oligonucleotide conjugates with improved cardiac exon skipping activity for DMD treatment. Mol. Ther. — Nucleic Acids 1, e38 23344180 10.1038/mtna.2012.30 PMC3438601

[78] Partridge T. A. (2013) The mdx mouse model as a surrogate for Duchenne muscular dystrophy. FEBS J. 280, 4177–4186 23551987 10.1111/febs.12267 PMC4147949

[79] Holland A., Henry M., Meleady P., Winkler C. K., Krautwald M., Brinkmeier H., and Ohlendieck K. (2015) Comparative label-free mass spectrometric analysis of mildly versus severely affected mdx mouse skeletal muscles identifies annexin, lamin, and vimentin as universal dystrophic markers. Molecules 20, 11317–11344 26102067 10.3390/molecules200611317 PMC6272583

[80] Percival J. M., Siegel M. P., Knowels G., and Marcinek D. J. (2013) Defects in mitochondrial localization and ATP synthesis in the mdx mouse model of Duchenne muscular dystrophy are not alleviated by PDE5 inhibition. Hum. Mol. Genet. 22, 153–167 23049075 10.1093/hmg/dds415 PMC3522404

[81] Bell E. L., Shine R. W., Dwyer P., Olson L., Truong J., Fredenburg R., Goddeeris M., Stickens D., and Tozzo E. (2019) PPARδ modulation rescues mitochondrial fatty acid oxidation defects in the mdx model of muscular dystrophy. Mitochondrion 46, 51–58 29458111 10.1016/j.mito.2018.02.006

[82] Gerhart-Hines Z., Rodgers J. T., Bare O., Lerin C., Kim S.-H., Mostoslavsky R., Alt F. W., Wu Z., and Puigserver P. (2007) Metabolic control of muscle mitochondrial function and fatty acid oxidation through SIRT1/PGC-1alpha. EMBO J. 26, 1913–1923 17347648 10.1038/sj.emboj.7601633 PMC1847661

[83] Rodgers J. T., Lerin C., Haas W., Gygi S. P., Spiegelman B. M., and Puigserver P. (2005) Nutrient control of glucose homeostasis through a complex of PGC-1alpha and SIRT1. Nature 434, 113–118 15744310 10.1038/nature03354

[84] Porter J. D., Khanna S., Kaminski H. J., Rao J. S., Merriam A. P., Richmonds C. R., Leahy P., Li J., Guo W., and Andrade F. H. (2002) A chronic inflammatory response dominates the skeletal muscle molecular signature in dystrophin-deficient mdx mice. Hum. Mol. Genet. 11, 263–272 11823445 10.1093/hmg/11.3.263

[85] Villalta S. A., Deng B., Rinaldi C., Wehling-Henricks M., and Tidball J. G. (2011) IFNγ promotes muscle damage in the mdx mouse model of Duchenne muscular dystrophy by suppressing M2 macrophage activation and inhibiting muscle cell proliferation. J. Immunol. Baltim. Md 1950 187, 5419–5428 10.4049/jimmunol.1101267 PMC3208069 22013114

[86] Monici M. C., Aguennouz M., Mazzeo A., Messina C., and Vita G. (2003) Activation of nuclear factor-kappaB in inflammatory myopathies and Duchenne muscular dystrophy. Neurology 60, 993–997 12654966 10.1212/01.wnl.0000049913.27181.51

[87] Acharyya S., Villalta S. A., Bakkar N., Bupha-Intr T., Janssen P. M. L., Carathers M., Li Z.-W., Beg A. A., Ghosh S., Sahenk Z., Weinstein M., Gardner K. L., Rafael-Fortney J. A., Karin M., Tidball J. G., Baldwin A. S., and Guttridge D. C. (2007) Interplay of IKK/NF-kappaB signaling in macrophages and myofibers promotes muscle degeneration in Duchenne muscular dystrophy. J. Clin. Invest. 117, 889–901 17380205 10.1172/JCI30556 PMC1821069

[88] Nelson D. E., Ihekwaba A. E. C., Elliott M., Johnson J. R., Gibney C. A., Foreman B. E., Nelson G., See V., Horton C. A., Spiller D. G., Edwards S. W., McDowell H. P., Unitt J. F., Sullivan E., Grimley R., Benson N., Broomhead D., Kell D. B., and White M. R. H. (2004) Oscillations in NF-kappaB signaling control the dynamics of gene expression. Science 306, 704–708 15499023 10.1126/science.1099962

[89] Kauppinen A., Suuronen T., Ojala J., Kaarniranta K., and Salminen A. (2013) Antagonistic crosstalk between NF-κB and SIRT1 in the regulation of inflammation and metabolic disorders. Cell. Signal. 25, 1939–1948 23770291 10.1016/j.cellsig.2013.06.007

[90] Alvarez-Guardia D., Palomer X., Coll T., Davidson M. M., Chan T. O., Feldman A. M., Laguna J. C., and Vázquez-Carrera M. (2010) The p65 subunit of NF-kappaB binds to PGC-1alpha, linking inflammation and metabolic disturbances in cardiac cells. Cardiovasc. Res. 87, 449–458 20211864 10.1093/cvr/cvq080

[91] Palomer X., Alvarez-Guardia D., Rodríguez-Calvo R., Coll T., Laguna J. C., Davidson M. M., Chan T. O., Feldman A. M., and Vázquez-Carrera M. (2009) TNF-alpha reduces PGC-1alpha expression through NF-kappaB and p38 MAPK leading to increased glucose oxidation in a human cardiac cell model. Cardiovasc. Res. 81, 703–712 19038972 10.1093/cvr/cvn327

[92] Dogra C., Changotra H., Wergedal J. E., and Kumar A. (2006) Regulation of phosphatidylinositol 3-kinase (PI3K)/Akt and nuclear factor-kappa B signaling pathways in dystrophin-deficient skeletal muscle in response to mechanical stretch. J. Cell. Physiol. 208, 575–585 16741926 10.1002/jcp.20696

[93] Glass D. J. (2010) PI3 kinase regulation of skeletal muscle hypertrophy and atrophy. Curr. Top. Microbiol. Immunol. 346, 267–278 20593312 10.1007/82_2010_78

[94] Sarbassov D. D., Guertin D. A., Ali S. M., and Sabatini D. M. (2005) Phosphorylation and regulation of Akt/PKB by the rictor-mTOR complex. Science 307, 1098–1101 15718470 10.1126/science.1106148

[95] Persad S., Attwell S., Gray V., Delcommenne M., Troussard A., Sanghera J., and Dedhar S. (2000) Inhibition of integrin-linked kinase (ILK) suppresses activation of protein kinase B/Akt and induces cell cycle arrest and apoptosis of PTEN-mutant prostate cancer cells. Proc. Natl. Acad. Sci. U S A 97, 3207–3212 10716737 10.1073/pnas.060579697 PMC16217

[96] Tian J., Chen J., Gao J., Li L., and Xie X. (2013) Resveratrol inhibits TNF-α-induced IL-1β, MMP-3 production in human rheumatoid arthritis fibroblast-like synoviocytes via modulation of PI3kinase/Akt pathway. Rheumatol. Int. 33, 1829–1835 23328930 10.1007/s00296-012-2657-0

[97] Li X., Monks B., Ge Q., and Birnbaum M. J. (2007) Akt/PKB regulates hepatic metabolism by directly inhibiting PGC-1alpha transcription coactivator. Nature 447, 1012–1016 17554339 10.1038/nature05861

[98] Handschin C., Kobayashi Y. M., Chin S., Seale P., Campbell K. P., and Spiegelman B. M. (2007) PGC-1alpha regulates the neuromuscular junction program and ameliorates Duchenne muscular dystrophy. Genes Dev. 21, 770–783 17403779 10.1101/gad.1525107 PMC1838529

[99] Pauly M., Daussin F., Burelle Y., Li T., Godin R., Fauconnier J., Koechlin-Ramonatxo C., Hugon G., Lacampagne A., Coisy-Quivy M., Liang F., Hussain S., Matecki S., and Petrof B. J. (2012) AMPK activation stimulates autophagy and ameliorates muscular dystrophy in the mdx mouse diaphragm. Am. J. Pathol. 181, 583–592 22683340 10.1016/j.ajpath.2012.04.004

[100] Hori Y. S., Kuno A., Hosoda R., Tanno M., Miura T., Shimamoto K., and Horio Y. (2011) Resveratrol ameliorates muscular pathology in the dystrophic mdx mouse, a model for Duchenne muscular dystrophy. J. Pharmacol. Exp. Ther. 338, 784–794 21652783 10.1124/jpet.111.183210

[101] Hammers D. W., Sleeper M. M., Forbes S. C., Coker C. C., Jirousek M. R., Zimmer M., Walter G. A., and Sweeney H. L. (2016) Disease-modifying effects of orally bioavailable NF-κB inhibitors in dystrophin-deficient muscle. JCI Insight. 1, e90341 28018975 10.1172/jci.insight.90341 PMC5161210

[102] Donovan J. M., Zimmer M., Offman E., Grant T., and Jirousek M. (2017) A novel NF-κB inhibitor, edasalonexent (CAT-1004), in development as a disease-modifying treatment for patients with Duchenne muscular dystrophy: phase 1 safety, pharmacokinetics, and pharmacodynamics in adult subjects. J. Clin. Pharmacol. 57, 627–639 28074489 10.1002/jcph.842 PMC5412838

[103] Grounds M. D., and Torrisi J. (2004) Anti-TNFα (Remicade®) therapy protects dystrophic skeletal muscle from necrosis. FASEB J. 18, 676–682 15054089 10.1096/fj.03-1024com

[104] Ermolova N. V., Martinez L., Vetrone S. A., Jordan M. C., Roos K. P., Sweeney H. L., and Spencer M. J. (2014) Long-term administration of the TNF blocking drug Remicade (cV1q) to mdx mice reduces skeletal and cardiac muscle fibrosis, but negatively impacts cardiac function. Neuromuscul. Disord. NMD 24, 583–595 24844454 10.1016/j.nmd.2014.04.006 PMC4122520

[105] Yu K., Yau Y. H., Sinha A., Tan T., Kickhoefer V. A., Rome L. H., Lee H., Shochat S. G., and Lim S. (2017) Modulation of the vault protein-protein interaction for tuning of molecular release. Sci. Rep 7, 14816 29093465 10.1038/s41598-017-12870-x PMC5665922

[106] Lee H. M., Joh J. W., Seo S.-R., Kim W.-T., Kim M. K., Choi H. S., Kim S. Y., Jang Y.-J., Sinn D. H., Choi G. S., Kim J. M., Kwon C. H. D., Chang H. J., Kim D. S., and Ryu C. J. (2017) Cell-surface major vault protein promotes cancer progression through harboring mesenchymal and intermediate circulating tumor cells in hepatocellular carcinomas. Sci. Rep 7, 13201 29038587 10.1038/s41598-017-13501-1 PMC5643512

[107] Xiang Z., Yuan W., Luo N., Wang Y., Tan K., Deng Y., Zhou X., Zhu C., Li Y., Liu M., Wu X., and Li Y. (2006) A novel human zinc finger protein ZNF540 interacts with MVP and inhibits transcriptional activities of the ERK signal pathway. Biochem. Biophys. Res. Commun. 347, 288–296 16815308 10.1016/j.bbrc.2006.06.076

[108] Das D., Wang Y. H., Hsieh C. Y., and Suzuki Y. J. (2016) Major vault protein regulates cell growth/survival signaling through oxidative modifications. Cell. Signal. 28, 12–18 26499037 10.1016/j.cellsig.2015.10.007 PMC4679458

