# Supplementary material for: Mutation-independent Proteomic Signatures of Pathological Progression in Murine Models of Duchenne Muscular Dystrophy
Source: Mol Cell Proteomics. 2020 Sep 28;19(12):2047–67. doi: 10.1074/mcp.RA120.002345 (PMC7710136; doi:10.1074/mcp.RA120.002345)
Supplement: supplemental Fig. S4 [file RA120.002345_index.html]

Supplement to Mutation-independent proteomic signatures of pathological progression in murine models of Duchenne muscular dystrophy | Molecular & Cellular Proteomics

## Supplemental Data

- File S1 - Supplementary Data
- Supplemental Data - Supplementary Tables, Figures and References
