# Supplementary material for: Mutation-independent Proteomic Signatures of Pathological Progression in Murine Models of Duchenne Muscular Dystrophy
Source: Mol Cell Proteomics. 2020 Sep 28;19(12):2047–67. doi: 10.1074/mcp.RA120.002345 (PMC7710136; doi:10.1074/mcp.RA120.002345)
Supplement: Supplemental Data [file 163442_0_supp_594816_qglj1l.pdf]

Supplemental Information

Antibody	Dilution	Product Code	Company
CAV3	1:500	ab2912	Abcam
MVP	1:500	ab152110	Abcam
PAK1	1:1,000	2602S	Cell Signaling Technology
GAPDH	1:5,000	14C10	Cell Signaling Technology

Table S1

List of antibodies used in this study.

Gene	Primer	Sequence
Cav3	Forward	CTACTCACTGTGTATCCGCAC
	Reverse	CTCACCTGACCTTTGATCCC
Mvp	Forward	TGACCAGGAGATCCGACTAG
	Reverse	CTTCAAAGTCCAGCAATGCC
Pak1	Forward	ACTCTTGATGTCCCTGTGAATG
	Reverse	CTGTATGGATGAAGGCCAGAT
Rpl10	Forward	TCATGTCCATCCGAACCAAG
	Reverse	GCATTAAACTTGGTGAAGCCC
Rplp0	Forward	AAGCAAAGGAAGAGTCGGAG
	Reverse	CCAGACCGGAGTTTTAAGAGAAG
Tbp	Forward	AAGAAAGGGAGAATCATGGACC
	Reverse	GAGTAAGTCCTGTGCCGTAAG

Table S2

List of RT-qPCR primers used in this study.

All sequences are 5′ to 3′.

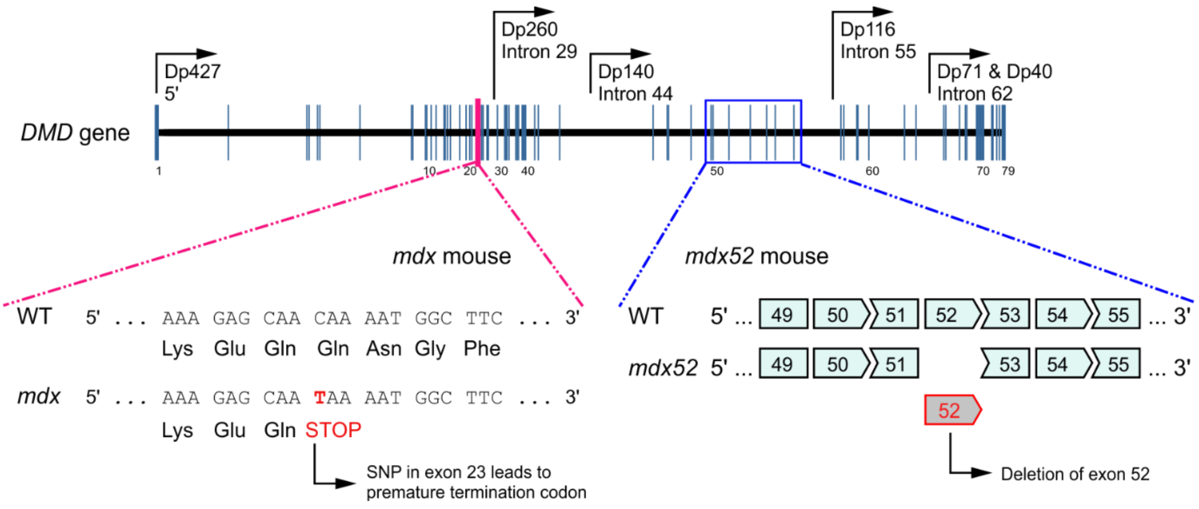
Dp427
5'
Dp260
Intron 29
Dp140
Intron 44
Dp116
Intron 55
Dp71 & Dp40
Intron 62
DMD gene
1
10
20
30
40
50
60
70
79
mdx mouse
WT 5' . . . AAA GAG CAA CAA AAT GGC TTC . . . 3'
Lys Glu Gln Gln Asn Gly Phe
mdx 5' . . . AAA GAG CAA TAA AAT GGC TTC . . . 3'
Lys Glu Gln STOP
SNP in exon 23 leads to premature termination codon
mdx52 mouse
WT 5' ... 49 50 51 52 53 54 55 ... 3'
mdx52 5' ... 49 50 51 53 54 55 ... 3'
52
Deletion of exon 52

Figure S1

Dystrophin mutations in the *mdx* and *mdx52* mouse models.

Schematic of the *Dmd* locus encoding the dystrophin protein. The location and details of mutations are indicated. The *mdx* mouse exhibits a point mutation in exon 23, leading to a premature stop codon. Conversely, the *mdx52* mouse carries a deletion of exon 52. Both mutations disrupt the translation reading frame of the largest dystrophin isoform (i.e. Dp427), while the *mdx52*-type mutation also disrupts other dystrophin isoforms.

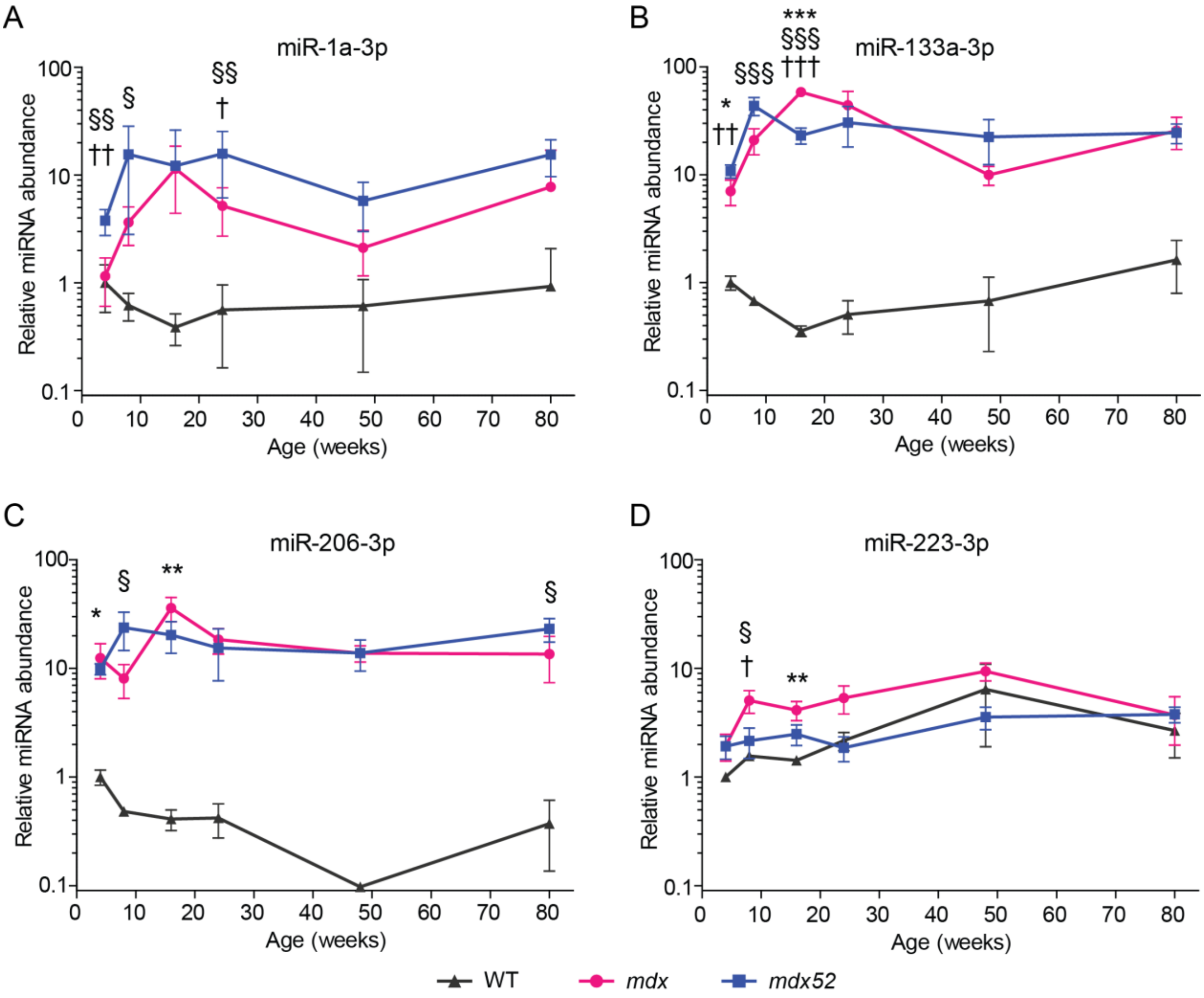
A
miR-1a-3p
B
miR-133a-3p
C
miR-206-3p
D
miR-223-3p
Relative miRNA abundance
Age (weeks)
WT
mdx
mdx52

Figure S2

Serum miRNA biomarker levels in *mdx* and *mdx52* mice.

Extracellular miRNA biomarker abundance was measured by small RNA TaqMan RT-qPCR in serum samples from WT, *mdx* and *mdx52* mice at 4, 8, 16, 24, 48 and 80 weeks of age for (A) miR-1a-3p, (B) miR-133a-3p, (C), miR-206-3p, and (D) miR-223-3p (an endogenous control which is not expected to change between groups). miRNA-of-interest abundance was normalized to an external synthetic control oligonucleotide (cel-miR-39). All values are mean±SEM, n=3. *P<0.05, **P<0.01 for comparisons between *mdx* and WT. §P<0.05, §§P<0.01 for comparisons between *mdx52* and WT. ††P<0.001 or comparisons between *mdx* and *mdx52*. Statistical analyses are one-way ANOVA at each time point, with Tukey or Games-Howell *post hoc* correction, as appropriate.

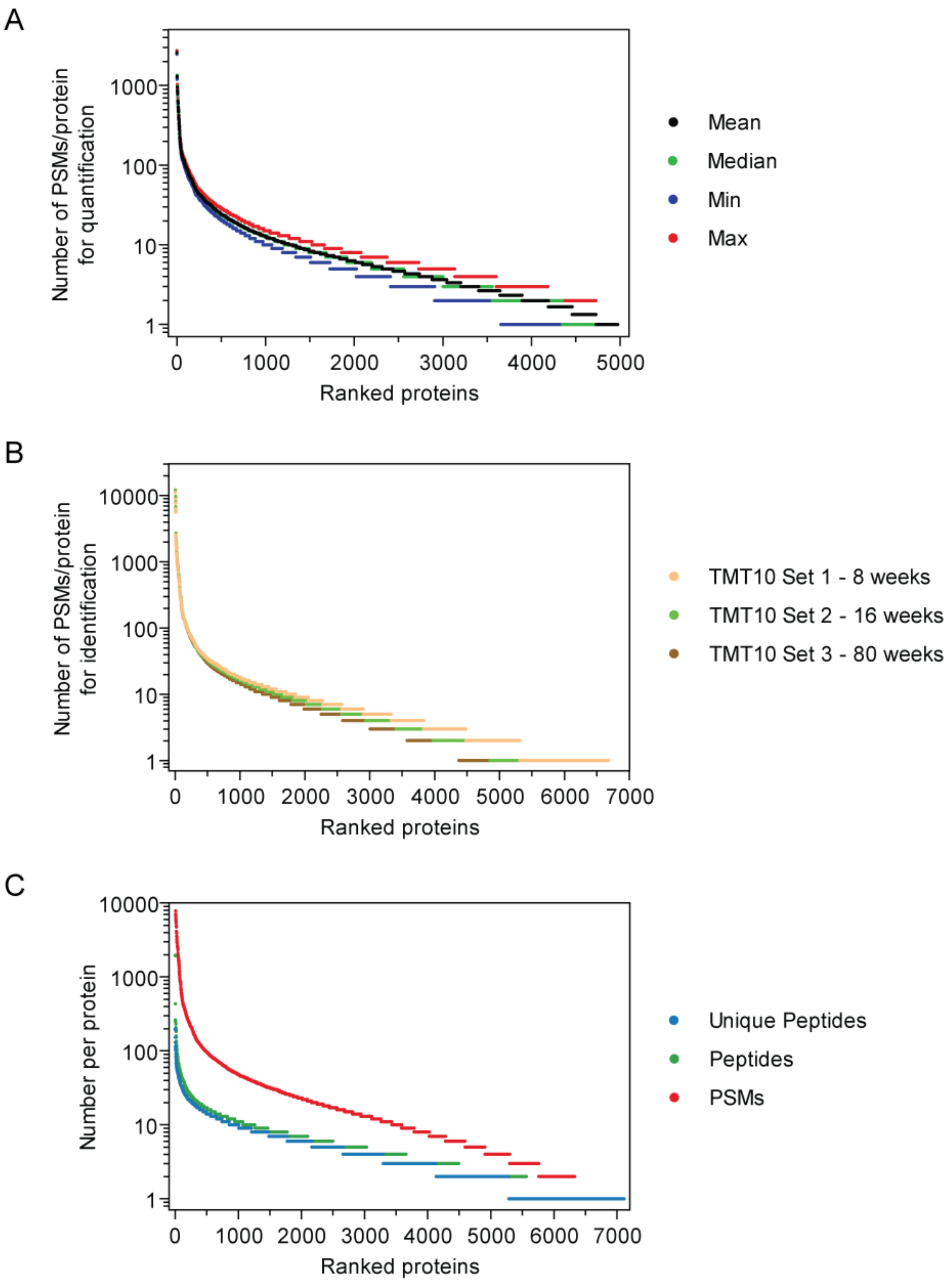
A
Number of PSMs/protein for quantification
1000
100
10
1
0
1000
2000
3000
4000
5000
Ranked proteins
Mean
Median
Min
Max
B
Number of PSMs/protein for identification
10000
1000
100
10
1
0
1000
2000
3000
4000
5000
6000
7000
Ranked proteins
TMT10 Set 1 - 8 weeks
TMT10 Set 2 - 16 weeks
TMT10 Set 3 - 80 weeks
C
Number per protein
10000
1000
100
10
1
0
1000
2000
3000
4000
5000
6000
7000
Ranked proteins
Unique Peptides
Peptides
PSMs

Figure S3

Robustness evaluation of proteomics peptide data.

(A) All 7,111 identified proteins ranked by the number of unique peptides per protein, number of peptides and peptide spectrum matches (PSMs) per protein for identification, across the 3 TMT10 sets. (B) Ranking of (PSMs) per protein for identification for each TMT set. (C) Ranking of the 4,974 proteins with TMT quantification across all 3 TMT10 sets that were used in the proteome analysis, based on the number of PSMs per protein for quantification. The protein quantification for this dataset is based on an average of 14 and a median of 5 PSMs/protein.

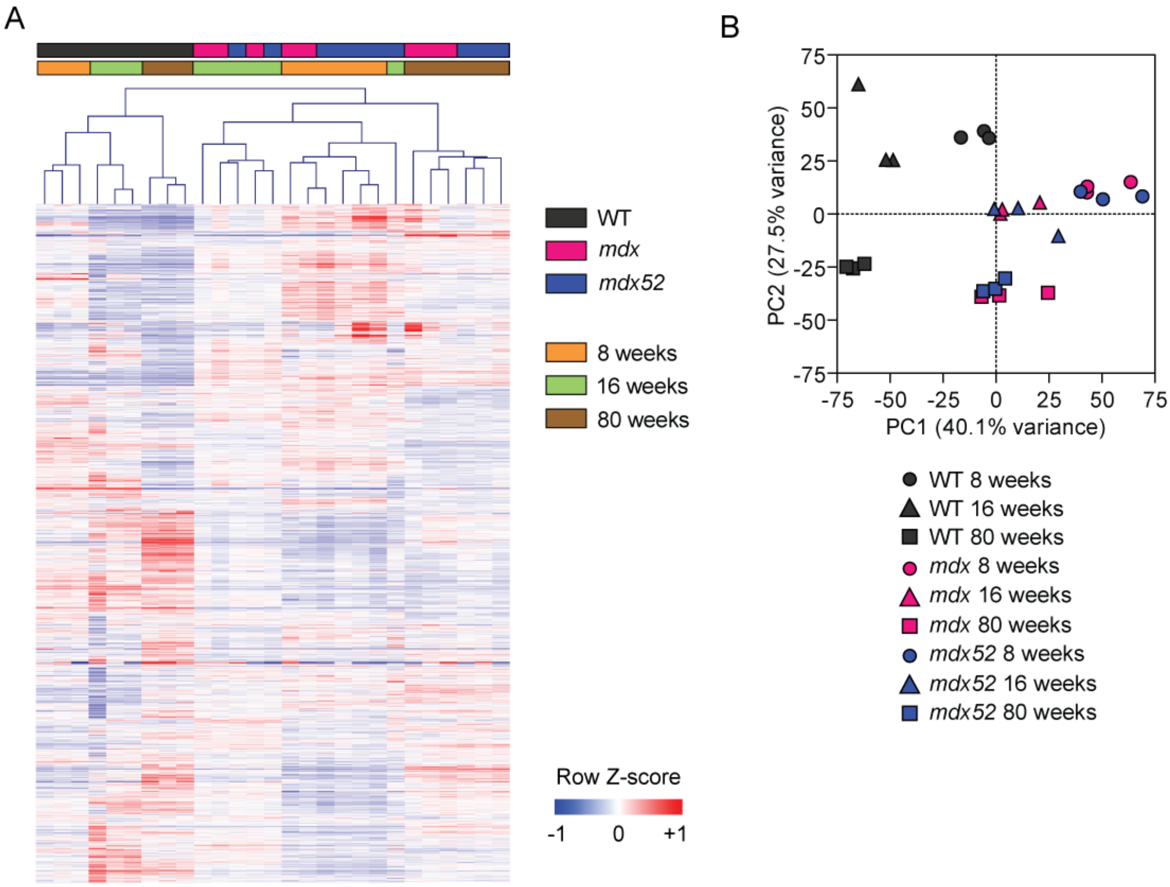
A
WT
mdx
mdx52
8 weeks
16 weeks
80 weeks
Row Z-score
-1
0
+1
B
PC2 (27.5% variance)
PC1 (40.1% variance)
75
50
25
0
-25
-50
-75
-75 -50 -25 0 25 50 75
WT 8 weeks
WT 16 weeks
WT 80 weeks
mdx 8 weeks
mdx 16 weeks
mdx 80 weeks
mdx52 8 weeks
mdx52 16 weeks
mdx52 80 weeks

Figure S4

Unsupervised quantitative proteomics analysis.

Quantitative proteomics analysis of TA muscles from WT (C57BL/6), *mdx* and *mdx52* mice at 8, 16 and 80 weeks of age (all n=3). All proteins that were quantified in all samples are visualized by (A) hierarchical clustering and heatmap, and (B) principal component analysis (PCA).

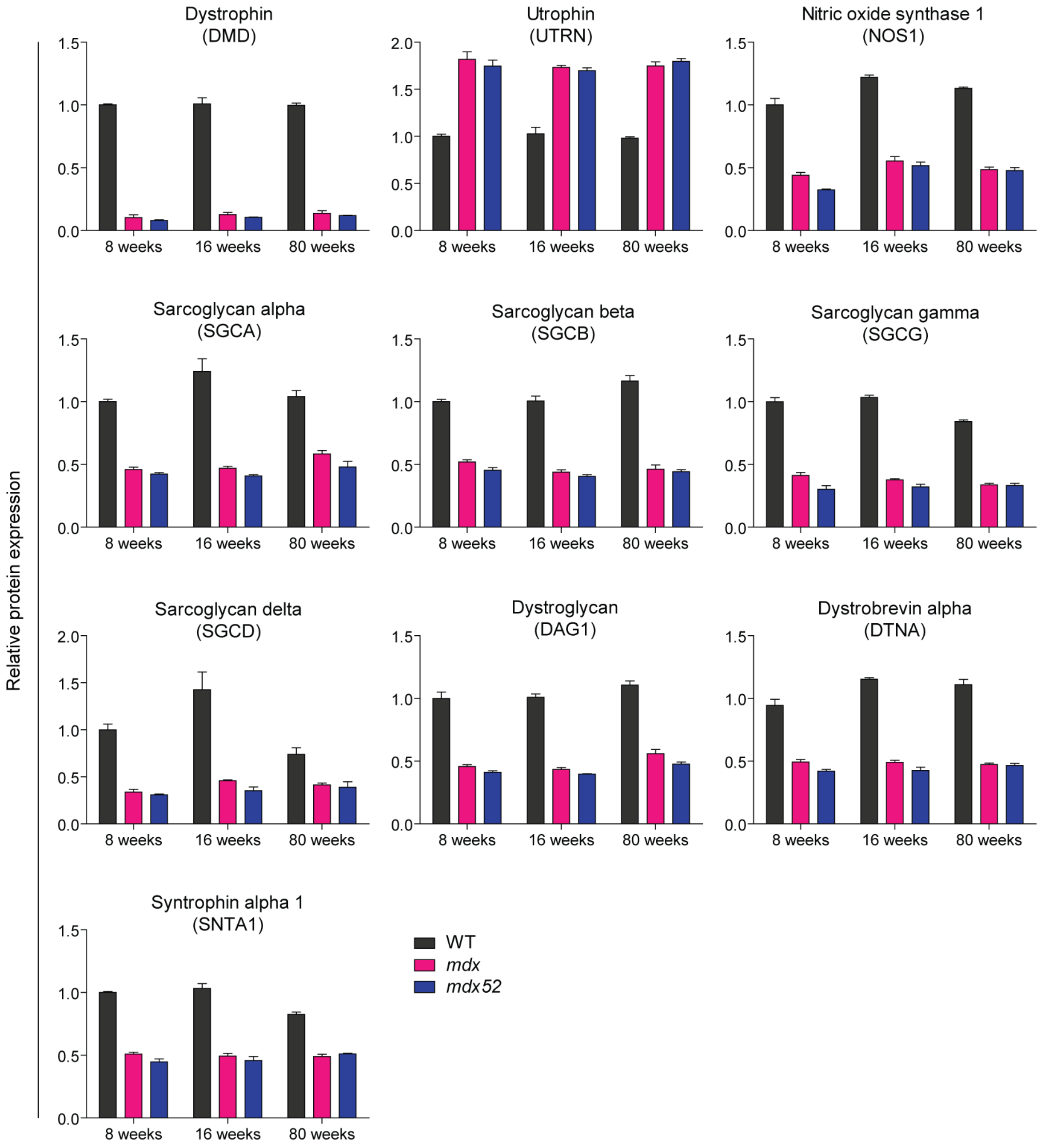
Relative protein expression
Dystrophin (DMD)
Utrophin (UTRN)
Nitric oxide synthase 1 (NOS1)
Sarcoglycan alpha (SGCA)
Sarcoglycan beta (SGCB)
Sarcoglycan gamma (SGCG)
Sarcoglycan delta (SGCD)
Dystroglycan (DAG1)
Dystrobrevin alpha (DTNA)
Syntrophin alpha 1 (SNTA1)
8 weeks
16 weeks
80 weeks
WT
mdx
mdx52

Figure S5

Protein expression of DAPC components.

Protein expression levels identified by HiRIEF-LC-MS/MS are shown for WT, *mdx* and *mdx52* samples at 8, 16 and 80 weeks of age. Values are mean+SEM, *n*=3.

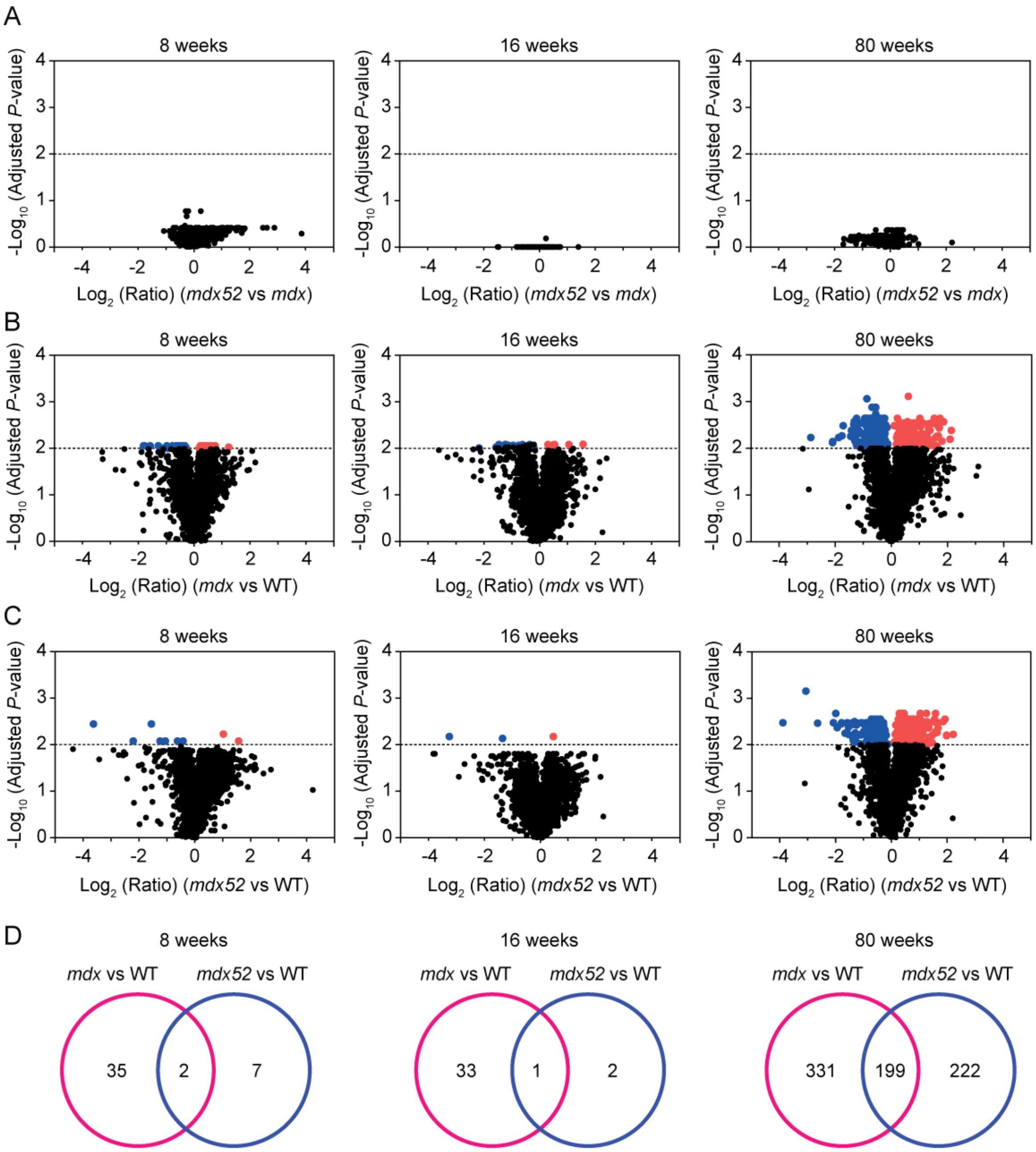

A
8 weeks
16 weeks
80 weeks
-Log10 (Adjusted P-value)
Log2 (Ratio) (mdx52 vs mdx)
B
Log2 (Ratio) (mdx vs WT)
C
Log2 (Ratio) (mdx52 vs WT)
D
mdx vs WT
mdx52 vs WT
35
2
7
33
1
2
331
199
222

Figure S6

Differentially expressed proteins between WT, *mdx* and *mdx52* at 8, 16 and 80 weeks of age.

Volcano plots showing differentially expressed proteins (adjusted $P<0.01$, *t*-test) when comparing (A) *mdx52* vs *mdx*, (B) *mdx* vs WT, or (C) *mdx52* vs WT at each age (8, 16, and 80 weeks). (D) Venn diagrams showing the overlap between differential expression calls in *mdx* vs WT and *mdx52* vs WT comparisons at each age.

A

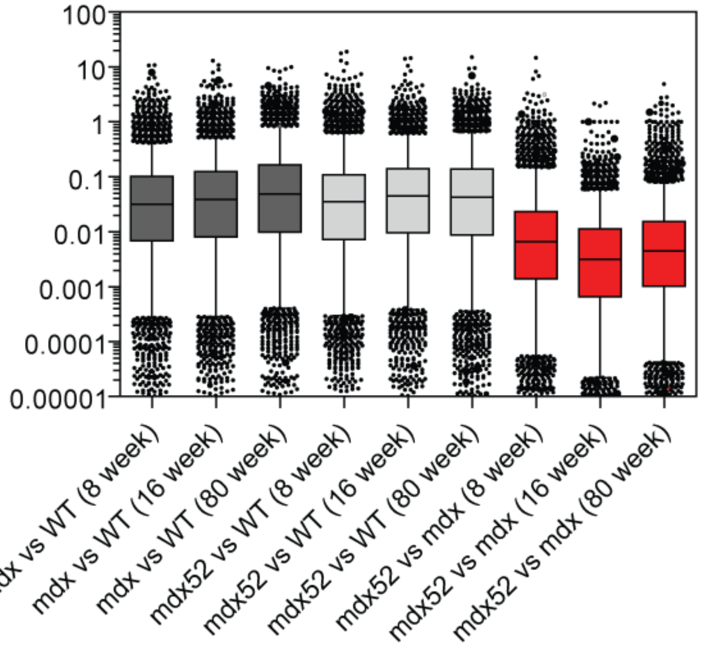
100
10
1
0.1
0.01
0.001
0.0001
0.00001
|fold change|
mdx vs WT (8 week)
mdx vs WT (16 week)
mdx vs WT (80 week)
mdx52 vs WT (8 week)
mdx52 vs WT (16 week)
mdx52 vs WT (80 week)
mdx52 vs mdx (8 week)
mdx52 vs mdx (16 week)
mdx52 vs mdx (80 week)

B

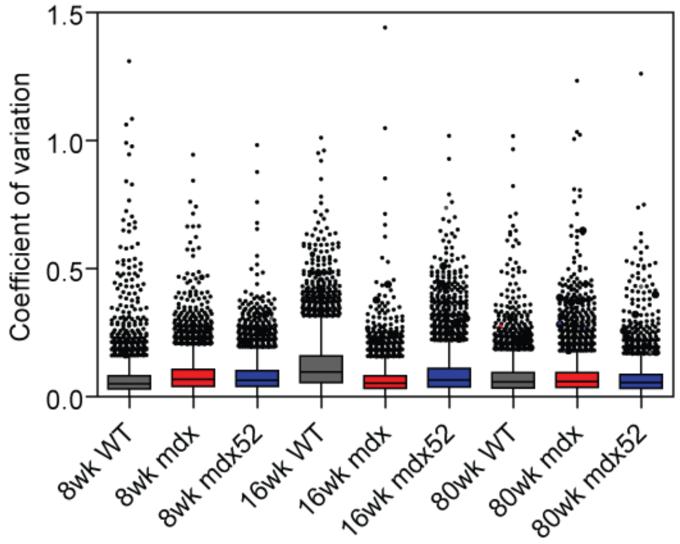
1.5
1.0
0.5
0.0
Coefficient of variation
8wk WT
8wk mdx
8wk mdx52
16wk WT
16wk mdx
16wk mdx52
80wk WT
80wk mdx
80wk mdx52

Figure S7

***mdx52* and *mdx* muscle proteomes are highly similar.**

(A) Absolute fold changes for *mdx* vs WT, *mdx52* vs WT and *mdx52* vs *mdx* comparisons. The distribution of fold changes indicates that *mdx52* and *mdx* proteomes are highly similar. (B) Coefficients of variation (CVs) for all groups. The *mdx52* samples exhibit similar, or lower CV values to other groups.

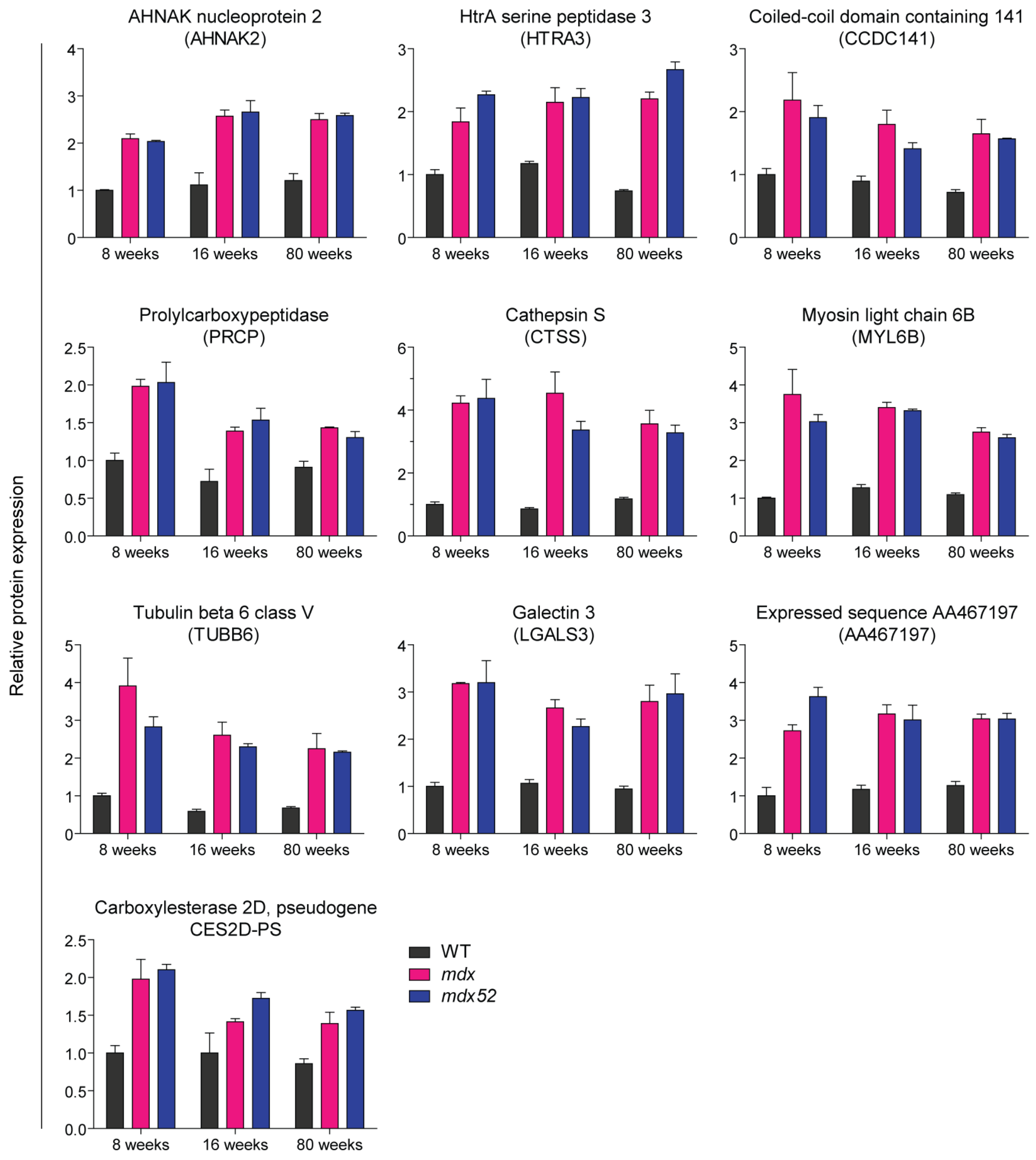

Relative protein expression
AHNAK nucleoprotein 2 (AHNAK2)
HtrA serine peptidase 3 (HTRA3)
Coiled-coil domain containing 141 (CCDC141)
Prolylcarboxypeptidase (PRCP)
Cathepsin S (CTSS)
Myosin light chain 6B (MYL6B)
Tubulin beta 6 class V (TUBB6)
Galectin 3 (LGALS3)
Expressed sequence AA467197 (AA467197)
Carboxylesterase 2D, pseudogene CES2D-PS
8 weeks
16 weeks
80 weeks
WT
mdx
mdx52

Figure S8

Top 10 up-regulated proteins in dystrophic muscle.

Statistically significant proteins (adjusted $P<0.01$, all Dystrophic vs all WT, *t*-test) were ranked by positive fold change and expression ratios are shown for all experimental groups in the case of the top 10 candidates. Protein expression levels identified by HiRIEF-LC-MS/MS are shown for WT, *mdx* and *mdx52* samples at 8, 16 and 80 weeks of age. Proteins that were not consistently expressed between *mdx* and *mdx52* were excluded from this analysis (i.e. MTHFD1L, FN1, LCP1, and PVR). Data for one additional top-scoring protein (PAK1) is shown in **Figure 7**. Values are mean+SEM, $n=3$.

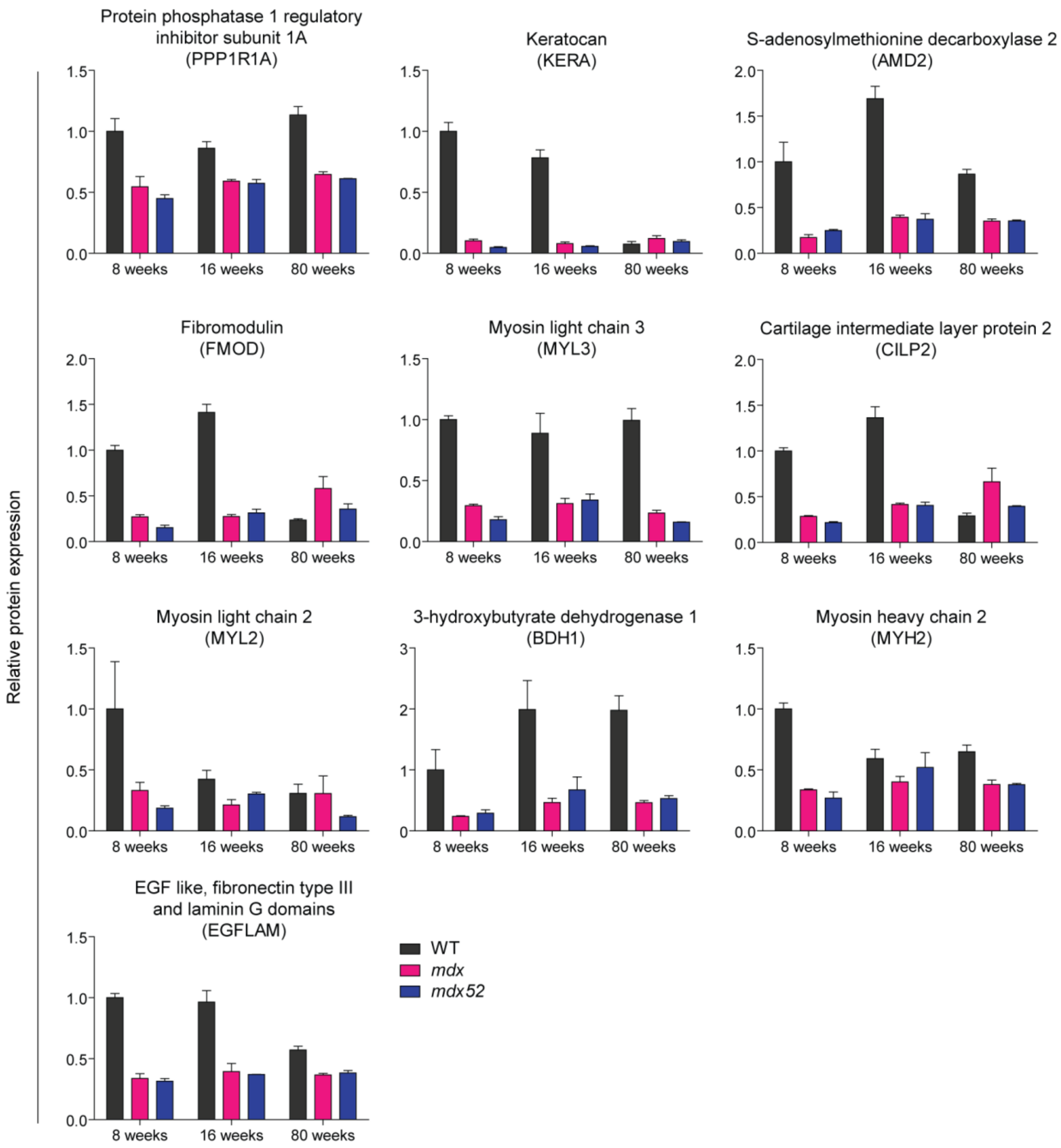
Relative protein expression
Protein phosphatase 1 regulatory inhibitor subunit 1A (PPP1R1A)
Keratocan (KERA)
S-adenosylmethionine decarboxylase 2 (AMD2)
Fibromodulin (FMOD)
Myosin light chain 3 (MYL3)
Cartilage intermediate layer protein 2 (CILP2)
Myosin light chain 2 (MYL2)
3-hydroxybutyrate dehydrogenase 1 (BDH1)
Myosin heavy chain 2 (MYH2)
EGF like, fibronectin type III and laminin G domains (EGFLAM)
8 weeks
16 weeks
80 weeks
WT
mdx
mdx52

Figure S9

Top 10 down-regulated proteins in dystrophic muscle.

Statistically significant proteins (adjusted $P<0.01$, all Dystrophic vs all WT, *t*-test) were ranked by negative fold change and expression ratios are shown for all experimental groups in the case of the top 10 candidates. Protein expression levels identified by HiRIEF-LC-MS/MS are shown for WT, *mdx* and *mdx52* samples at 8, 16 and 80 weeks of age. Known DAPC components were excluded from this analysis as these are shown in **Figure S4**. Values are mean+SEM, $n=3$.

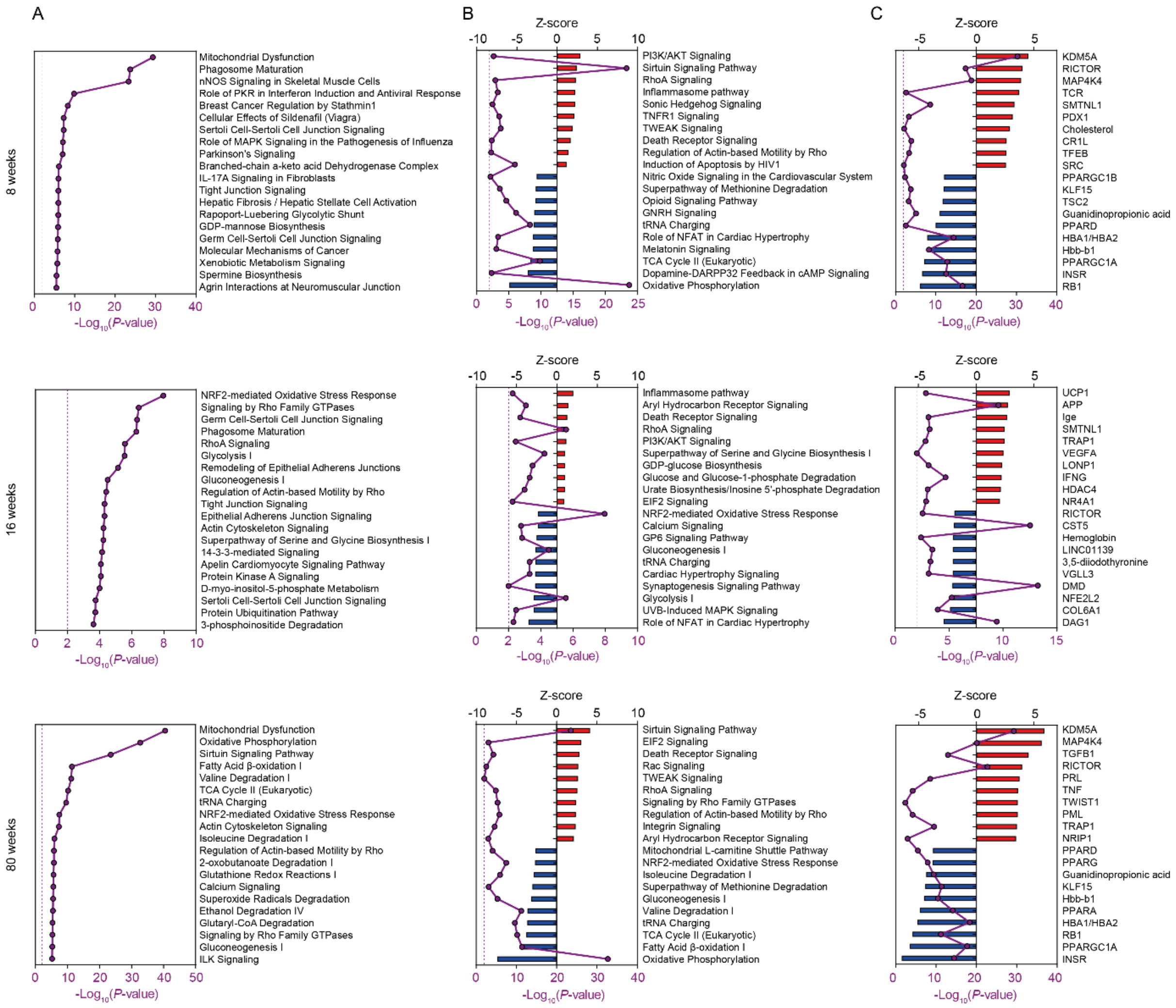
A
B
C
Z-score
8 weeks
Mitochondrial Dysfunction
Phagosome Maturation
nNOS Signaling in Skeletal Muscle Cells
Role of PKR in Interferon Induction and Antiviral Response
Breast Cancer Regulation by Stathmin1
Cellular Effects of Sildenafil (Viagra)
Sertoli Cell-Sertoli Cell Junction Signaling
Role of MAPK Signaling in the Pathogenesis of Influenza
Parkinson's Signaling
Branched-chain a-keto acid Dehydrogenase Complex
IL-17A Signaling in Fibroblasts
Tight Junction Signaling
Hepatic Fibrosis / Hepatic Stellate Cell Activation
Rapoport-Luebering Glycolytic Shunt
GDP-mannose Biosynthesis
Germ Cell-Sertoli Cell Junction Signaling
Molecular Mechanisms of Cancer
Xenobiotic Metabolism Signaling
Spermine Biosynthesis
Agrin Interactions at Neuromuscular Junction
-Log10(P-value)
PI3K/AKT Signaling
Sirtuin Signaling Pathway
RhoA Signaling
Inflammasome pathway
Sonic Hedgehog Signaling
TNFR1 Signaling
TWEAK Signaling
Death Receptor Signaling
Regulation of Actin-based Motility by Rho
Induction of Apoptosis by HIV1
Nitric Oxide Signaling in the Cardiovascular System
Superpathway of Methionine Degradation
Opioid Signaling Pathway
GNRH Signaling
tRNA Charging
Role of NFAT in Cardiac Hypertrophy
Melatonin Signaling
TCA Cycle II (Eukaryotic)
Dopamine-DARPP32 Feedback in cAMP Signaling
Oxidative Phosphorylation
KDM5A
RICTOR
MAP4K4
TCR
SMTNL1
PDX1
Cholesterol
CR1L
TFEB
SRC
PPARGC1B
KLF15
TSC2
Guanidinopropionic acid
PPARD
HBA1/HBA2
Hbb-b1
PPARGC1A
INSR
RB1
16 weeks
NRF2-mediated Oxidative Stress Response
Signaling by Rho Family GTPases
Germ Cell-Sertoli Cell Junction Signaling
Phagosome Maturation
RhoA Signaling
Glycolysis I
Remodeling of Epithelial Adherens Junctions
Gluconeogenesis I
Regulation of Actin-based Motility by Rho
Tight Junction Signaling
Epithelial Adherens Junction Signaling
Actin Cytoskeleton Signaling
Superpathway of Serine and Glycine Biosynthesis I
14-3-3-mediated Signaling
Apelin Cardiomyocyte Signaling Pathway
Protein Kinase A Signaling
D-myo-inositol-5-phosphate Metabolism
Sertoli Cell-Sertoli Cell Junction Signaling
Protein Ubiquitination Pathway
3-phosphoinositide Degradation
Inflammasome pathway
Aryl Hydrocarbon Receptor Signaling
Death Receptor Signaling
RhoA Signaling
PI3K/AKT Signaling
Superpathway of Serine and Glycine Biosynthesis I
GDP-glucose Biosynthesis
Glucose and Glucose-1-phosphate Degradation
Urate Biosynthesis/Inosine 5'-phosphate Degradation
EIF2 Signaling
NRF2-mediated Oxidative Stress Response
Calcium Signaling
GP6 Signaling Pathway
Gluconeogenesis I
tRNA Charging
Cardiac Hypertrophy Signaling
Synaptogenesis Signaling Pathway
Glycolysis I
UVB-Induced MAPK Signaling
Role of NFAT in Cardiac Hypertrophy
UCP1
APP
Ige
SMTNL1
TRAP1
VEGFA
LONP1
IFNG
HDAC4
NR4A1
RICTOR
CST5
Hemoglobin
LINC01139
3,5-diiodothyronine
VGLL3
DMD
NFE2L2
COL6A1
DAG1
80 weeks
Mitochondrial Dysfunction
Oxidative Phosphorylation
Sirtuin Signaling Pathway
Fatty Acid β-oxidation I
Valine Degradation I
TCA Cycle II (Eukaryotic)
tRNA Charging
NRF2-mediated Oxidative Stress Response
Actin Cytoskeleton Signaling
Isoleucine Degradation I
Regulation of Actin-based Motility by Rho
2-oxobutanoate Degradation I
Glutathione Redox Reactions I
Calcium Signaling
Superoxide Radicals Degradation
Ethanol Degradation IV
Glutaryl-CoA Degradation
Signaling by Rho Family GTPases
Gluconeogenesis I
ILK Signaling
Sirtuin Signaling Pathway
EIF2 Signaling
Death Receptor Signaling
Rac Signaling
TWEAK Signaling
RhoA Signaling
Signaling by Rho Family GTPases
Regulation of Actin-based Motility by Rho
Integrin Signaling
Aryl Hydrocarbon Receptor Signaling
Mitochondrial L-carnitine Shuttle Pathway
NRF2-mediated Oxidative Stress Response
Isoleucine Degradation I
Superpathway of Methionine Degradation
Gluconeogenesis I
Valine Degradation I
tRNA Charging
TCA Cycle II (Eukaryotic)
Fatty Acid β-oxidation I
Oxidative Phosphorylation
KDM5A
MAP4K4
TGFB1
RICTOR
PRL
TNF
TWIST1
PML
TRAP1
NRIP1
PPARD
PPARG
Guanidinopropionic acid
KLF15
Hbb-b1
PPARA
HBA1/HBA2
RB1
PPARGC1A
INSR

Figure S10

Pathway analysis of dystrophic muscle at 8, 16 and 80 weeks of age.

(A) Top 20 significantly (P<0.01) enriched pathways identified in dystrophic muscle at each age identified using IPA. (B) Top 10 up- and down-regulated canonical pathways where a change in activation state (i.e. Z-score) was called. (C) Top 10 up- and down-regulated putative upstream regulators in dystrophic muscle at each age identified using IPA. Canonical pathways and upstream regulators are ranked by Z-score (positive values indicate activation and negative values indicate inhibition of the predicted regulator). Solid purple lines indicate P-value. Dotted purple lines indicate the P=0.01 significance threshold.

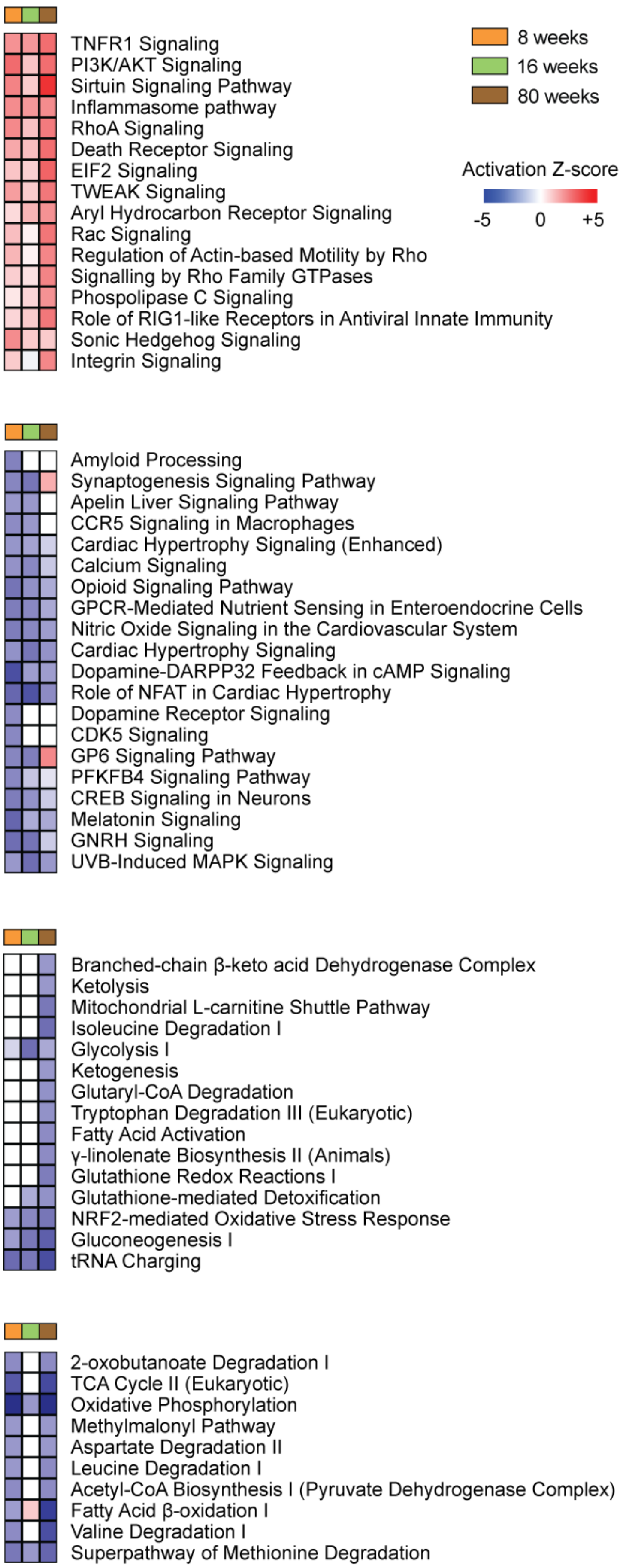
8 weeks
16 weeks
80 weeks
Activation Z-score
-5
0
+5
TNFR1 Signaling
PI3K/AKT Signaling
Sirtuin Signaling Pathway
Inflammasome pathway
RhoA Signaling
Death Receptor Signaling
EIF2 Signaling
TWEAK Signaling
Aryl Hydrocarbon Receptor Signaling
Rac Signaling
Regulation of Actin-based Motility by Rho
Signalling by Rho Family GTPases
Phospolipase C Signaling
Role of RIG1-like Receptors in Antiviral Innate Immunity
Sonic Hedgehog Signaling
Integrin Signaling
Amyloid Processing
Synaptogenesis Signaling Pathway
Apelin Liver Signaling Pathway
CCR5 Signaling in Macrophages
Cardiac Hypertrophy Signaling (Enhanced)
Calcium Signaling
Opioid Signaling Pathway
GPCR-Mediated Nutrient Sensing in Enteroendocrine Cells
Nitric Oxide Signaling in the Cardiovascular System
Cardiac Hypertrophy Signaling
Dopamine-DARPP32 Feedback in cAMP Signaling
Role of NFAT in Cardiac Hypertrophy
Dopamine Receptor Signaling
CDK5 Signaling
GP6 Signaling Pathway
PFKFB4 Signaling Pathway
CREB Signaling in Neurons
Melatonin Signaling
GNRH Signaling
UVB-Induced MAPK Signaling
Branched-chain β-keto acid Dehydrogenase Complex
Ketolysis
Mitochondrial L-carnitine Shuttle Pathway
Isoleucine Degradation I
Glycolysis I
Ketogenesis
Glutaryl-CoA Degradation
Tryptophan Degradation III (Eukaryotic)
Fatty Acid Activation
γ-linolenate Biosynthesis II (Animals)
Glutathione Redox Reactions I
Glutathione-mediated Detoxification
NRF2-mediated Oxidative Stress Response
Gluconeogenesis I
tRNA Charging
2-oxobutanoate Degradation I
TCA Cycle II (Eukaryotic)
Oxidative Phosphorylation
Methylmalonyl Pathway
Aspartate Degradation II
Leucine Degradation I
Acetyl-CoA Biosynthesis I (Pyruvate Dehydrogenase Complex)
Fatty Acid β-oxidation I
Valine Degradation I
Superpathway of Methionine Degradation

Figure S11

Perturbed canonical pathways in dystrophic muscle throughout disease progression.

IPA was utilized to identify canonical pathways that were perturbed in dystrophic muscle at 8, 16, and 80 weeks of age. All statistically significantly ($P<0.01$) canonical pathways with a corresponding activation state score are shown with *k*-means clustering used to generate 4 clusters according to their observed patterns of activation. Values are activation Z-scores whereby red indicates activation and blue indicates inhibition.

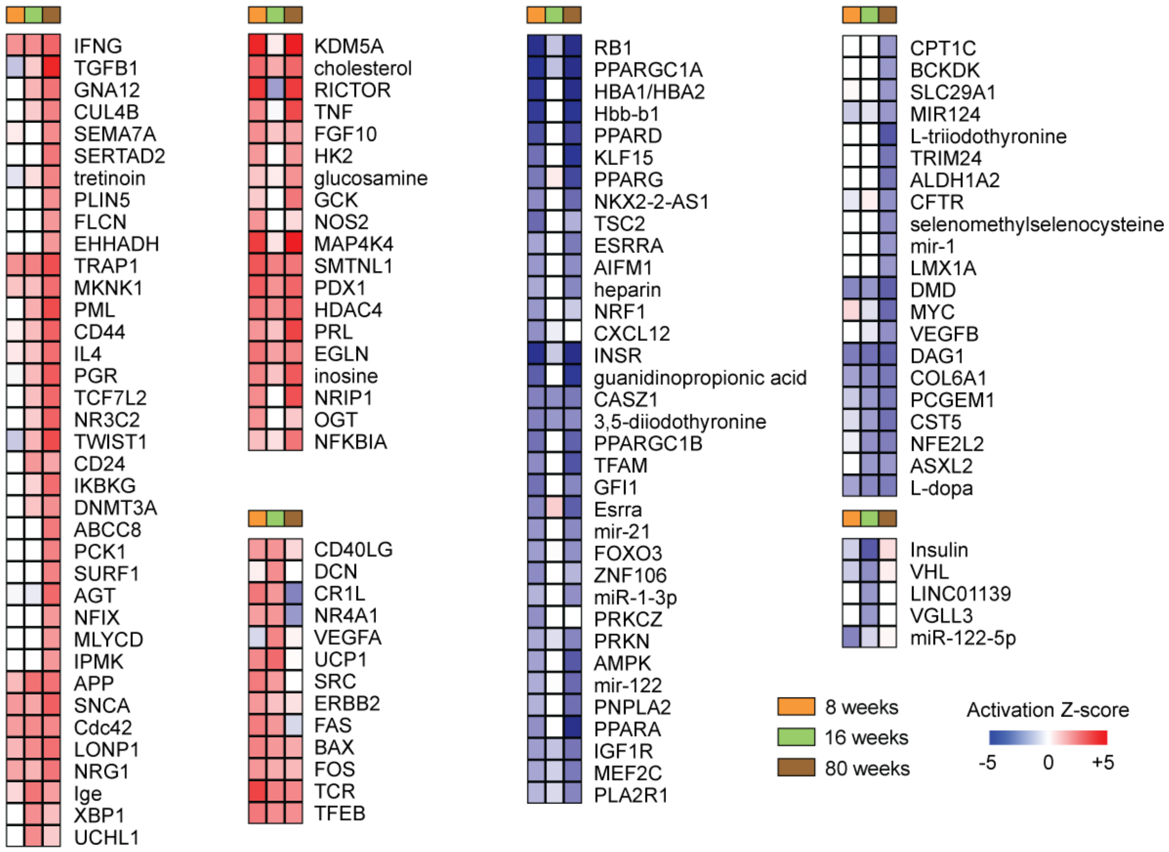
IFNG
TGFB1
GNA12
CUL4B
SEMA7A
SERTAD2
tretinoin
PLIN5
FLCN
EHHADH
TRAP1
MKNK1
PML
CD44
IL4
PGR
TCF7L2
NR3C2
TWIST1
CD24
IKBKG
DNMT3A
ABCC8
PCK1
SURF1
AGT
NFIX
MLYCD
IPMK
APP
SNCA
Cdc42
LONP1
NRG1
Ige
XBP1
UCHL1
KDM5A
cholesterol
RICTOR
TNF
FGF10
HK2
glucosamine
GCK
NOS2
MAP4K4
SMTNL1
PDX1
HDAC4
PRL
EGLN
inosine
NRIP1
OGT
NFKBIA
CD40LG
DCN
CR1L
NR4A1
VEGFA
UCP1
SRC
ERBB2
FAS
BAX
FOS
TCR
TFEB
RB1
PPARGC1A
HBA1/HBA2
Hbb-b1
PPARD
KLF15
PPARG
NKX2-2-AS1
TSC2
ESRRA
AIFM1
heparin
NRF1
CXCL12
INSR
guanidinopropionic acid
CASZ1
3,5-diiodothyronine
PPARGC1B
TFAM
GFI1
Esrra
mir-21
FOXO3
ZNF106
miR-1-3p
PRKCZ
PRKN
AMPK
mir-122
PNPLA2
PPARA
IGF1R
MEF2C
PLA2R1
CPT1C
BCKDK
SLC29A1
MIR124
L-triiodothyronine
TRIM24
ALDH1A2
CFTR
selenomethylselenocysteine
mir-1
LMX1A
DMD
MYC
VEGFB
DAG1
COL6A1
PCGEM1
CST5
NFE2L2
ASXL2
L-dopa
Insulin
VHL
LINC01139
VGLL3
miR-122-5p
8 weeks
16 weeks
80 weeks
Activation Z-score
-5
0
+5

Figure S12

Perturbed upstream regulators in dystrophic muscle throughout disease progression.

IPA was utilized to identify upstream regulators that were predicted to be perturbed in dystrophic muscle at 8, 16, and 80 weeks of age. All statistically significantly (P<0.01) regulated regulators are shown with *k*-means clustering used to generate 6 clusters according to their observed patterns of activation. Values are activation Z-scores whereby red indicates activation and blue indicates inhibition.

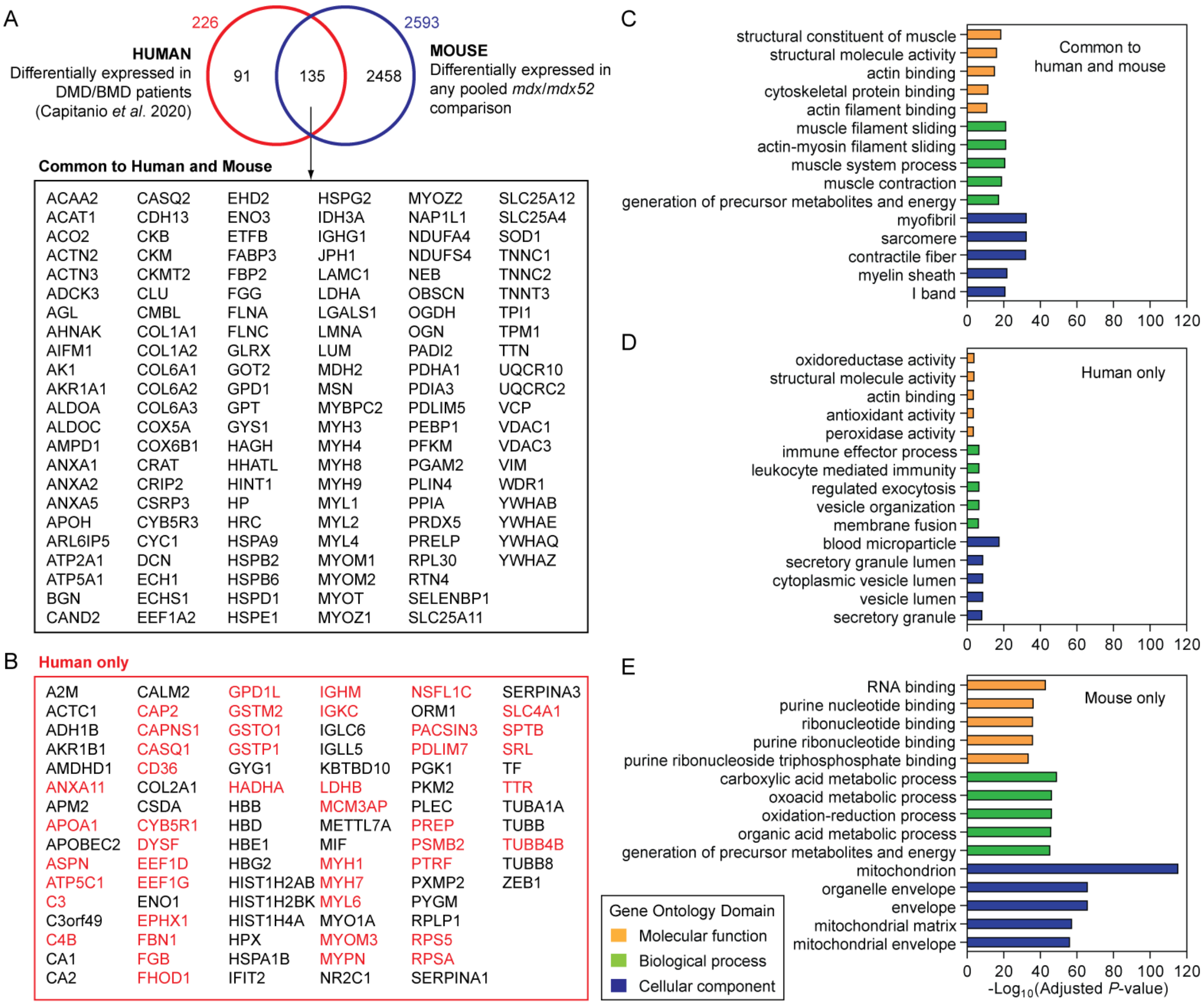
A
226
2593
HUMAN
Differentially expressed in DMD/BMD patients (Capitanio et al. 2020)
91
135
2458
MOUSE
Differentially expressed in any pooled mdx/mdx52 comparison
Common to Human and Mouse
ACAA2 CASQ2 EHD2 HSPG2 MYOZ2 SLC25A12
ACAT1 CDH13 ENO3 IDH3A NAP1L1 SLC25A4
ACO2 CKB ETFB IGHG1 NDUFA4 SOD1
ACTN2 CKM FABP3 JPH1 NDUFS4 TNNC1
ACTN3 CKMT2 FBP2 LAMC1 NEB TNNC2
ADCK3 CLU FGG LDHA OBSCN TNNT3
AGL CMBL FLNA LGALS1 OGDH TPI1
AHNAK COL1A1 FLNC LMNA OGN TPM1
AIFM1 COL1A2 GLRX LUM PADI2 TTN
AK1 COL6A1 GOT2 MDH2 PDHA1 UQCR10
AKR1A1 COL6A2 GPD1 MSN PDIA3 UQCRC2
ALDOA COL6A3 GPT MYBPC2 PDLIM5 VCP
ALDOC COX5A GYS1 MYH3 PEBP1 VDAC1
AMPD1 COX6B1 HAGH MYH4 PFKM VDAC3
ANXA1 CRAT HHATL MYH8 PGAM2 VIM
ANXA2 CRIP2 HINT1 MYH9 PLIN4 WDR1
ANXA5 CSRP3 HP MYL1 PPIA YWHAB
APOH CYB5R3 HRC MYL2 PRDX5 YWHAE
ARL6IP5 CYC1 HSPA9 MYL4 PRELP YWHAQ
ATP2A1 DCN HSPB2 MYOM1 RPL30 YWHAZ
ATP5A1 ECH1 HSPB6 MYOM2 RTN4
BGN ECHS1 HSPD1 MYOT SELENBP1
CAND2 EEF1A2 HSPE1 MYOZ1 SLC25A11
B
Human only
A2M CALM2 GPD1L IGHM NSFL1C SERPINA3
ACTC1 CAP2 GSTM2 IGKC ORM1 SLC4A1
ADH1B CAPNS1 GSTO1 IGLC6 PACSIN3 SPTB
AKR1B1 CASQ1 GSTP1 IGLL5 PDLIM7 SRL
AMDHD1 CD36 GYG1 KBTBD10 PGK1 TF
ANXA11 COL2A1 HADHA LDHB PKM2 TTR
APM2 CSDA HBB MCM3AP PLEC TUBA1A
APOA1 CYB5R1 HBD METTL7A PREP TUBB
APOBEC2 DYSF HBE1 MIF PSMB2 TUBB4B
ASPN EEF1D HBG2 MYH1 PTRF TUBB8
ATP5C1 EEF1G HIST1H2AB MYH7 PXMP2 ZEB1
C3 ENO1 HIST1H2BK MYL6 PYGM
C3orf49 EPHX1 HIST1H4A MYO1A RPLP1
C4B FBN1 HPX MYOM3 RPS5
CA1 FGB HSPA1B MYPN RPSA
CA2 FHOD1 IFIT2 NR2C1 SERPINA1
C
Common to human and mouse
structural constituent of muscle
structural molecule activity
actin binding
cytoskeletal protein binding
actin filament binding
muscle filament sliding
actin-myosin filament sliding
muscle system process
muscle contraction
generation of precursor metabolites and energy
myofibril
sarcomere
contractile fiber
myelin sheath
I band
0 20 40 60 80 100 120
D
Human only
oxidoreductase activity
structural molecule activity
actin binding
antioxidant activity
peroxidase activity
immune effector process
leukocyte mediated immunity
regulated exocytosis
vesicle organization
membrane fusion
blood microparticle
secretory granule lumen
cytoplasmic vesicle lumen
vesicle lumen
secretory granule
0 20 40 60 80 100 120
E
Mouse only
RNA binding
purine nucleotide binding
ribonucleotide binding
purine ribonucleotide binding
purine ribonucleoside triphosphate binding
carboxylic acid metabolic process
oxoacid metabolic process
oxidation-reduction process
organic acid metabolic process
generation of precursor metabolites and energy
mitochondrion
organelle envelope
envelope
mitochondrial matrix
mitochondrial envelope
0 20 40 60 80 100 120
-Log10(Adjusted P-value)
Gene Ontology Domain
Molecular function
Biological process
Cellular component

Figure S13

Comparison of dystrophic mouse and DMD/BMD patient muscle proteomes. (A) Venn diagram showing overlap between differentially expressed protein in human DMD and BMD patients relative to healthy controls in the study by Capatanio *et al.* (1) and differentially expressed proteins in any of the dystrophic vs wild-type comparisons reported in the present study. The 135 commonly changed proteins are listed. (B) Proteins that were only identified as being differentially expressed in human dystrophic muscle are listed. Proteins highlighted in red were detected in our analyses, but not found to be differentially expressed. The lists of proteins were analysed using gene list enrichment analysis for (C) proteins common to both human and mouse dystrophic muscle, (D) those unique to the human data, and (C) those unique to the mouse data described here.

Supplementary References

1. Capitanio, D., Moriggi, M., Torretta, E., Barbacini, P., De Palma, S., Viganò, A., Lochmüller, H., Muntoni, F., Ferlini, A., Mora, M., and Gelfi, C. (2020) Comparative proteomic analyses of Duchenne muscular dystrophy and Becker muscular dystrophy muscles: changes contributing to preserve muscle function in Becker muscular dystrophy patients. *J Cachexia Sarcopenia Muscle* 11, 547–563
